# Probing Relaxation Dynamics in Five‐Coordinate Dysprosium Single‐Molecule Magnets

**DOI:** 10.1002/chem.202001235

**Published:** 2020-06-03

**Authors:** Vijay S. Parmar, Fabrizio Ortu, Xiaozhou Ma, Nicholas F. Chilton, Rodolphe Clérac, David P. Mills, Richard E. P. Winpenny

**Affiliations:** ^1^ Department of Chemistry The University of Manchester Oxford Road Manchester M13 9PL UK; ^2^ CNRS Centre de Recherche Paul Pascal Univ. Bordeaux, CRPP, UMR 5031 33600 Pessac France

**Keywords:** aryloxides, lanthanides, magnetic anisotropy, paramagnetic relaxation, single-molecule magnets

## Abstract

A new family of five‐coordinate lanthanide single‐molecule magnets (Ln SMMs) [Dy(Mes*O)_2_(THF)_2_X] (Mes*=2,4,6‐tri‐tert‐butylphenyl; X=Cl, **1**; Br, **2**; I, **3**) is reported with energy barriers to magnetic reversal >1200 K. The five‐coordinate Dy^III^ ions have distorted square pyramidal geometries, with halide anions on the apex, and two Mes*O ligands mutually *trans*‐ to each other, and the two THF molecules forming the second *trans*‐ pair. These geometrical features lead to a large magnetic anisotropy in these complexes along the *trans*‐Mes*O direction. QTM and Raman relaxation times are enhanced by varying the apex halide from Cl to Br to I, or by dilution in a diamagnetic yttrium analogue.

## Graphical abstract

**Halide influence**: A new family of five‐coordinate lanthanide‐based single‐molecule magnets, [Dy(Mes*O)_2_(THF)_2_X] (Mes*=2,4,6‐tri‐tert‐butylphenyl), (X=Cl, Br, I) is reported. While the influence of the different halides on the quantum tunnelling of magnetization and Raman processes is clearly established, the Orbach relaxation is independent of the halide substitution.

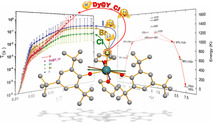

Single‐molecule magnets (SMMs) show slow relaxation of magnetisation under certain conditions; these have received huge interest in the last 25 years due to their potential applications in quantum computing, molecular spintronics and ultra‐high‐density storage.^1^, ^2^ For technological applications, it is important to determine the highest temperature at which a SMM can retain its magnetisation,^1^ and to understand the multiple relaxation mechanisms, which can be involved in their magnetic dynamics. Lanthanide (Ln) SMMs have provided some promising candidates since their discovery in 2003.^3^, ^4^ The design criteria to synthesise Ln SMMs,^5^ with a single Dy^III^ centre in a highly axial ligand field environment to generate large magnetic anisotropy and to stabilise the highest *m_J_*=±15/2 spin state of Dy^III^ as the ground state, has led to Ln SMMs with magnetisation dynamics dominated by an Orbach‐like (thermally activated) relaxation process, with very high values of the activation energy (Δ).^6^, ^7^, ^8^, ^9^, ^10^ Two of the recent high‐performing SMMs, [Dy(O*t*Bu)_2_(py)_5_][BPh_4_] (**4**)6a and [Dy(Cp^ttt^)_2_][B(C_6_F_5_)_4_] (**5**, Cp^ttt^, C_5_H_2_ *t*Bu_3_‐1,2,4)^7^ have similar Δ values (1815 K and 1760 K, respectively) but very different *T*_B_^100s^ (12 K in **4** and 53 K in **5**; here we use *T*_B_^100s^ to define the temperature at which the magnetic relaxation time is 100 s in zero field). The reasons behind the significant difference in *T*_B_^100s^, despite having comparable energy barriers, can be attributed to the differences in the other relaxation processes involved, for example, Raman and quantum tunnelling of magnetization (QTM), and therefore it is necessary to understand these mechanisms in detail to design higher‐performing SMMs.^11^, ^12^, ^13^, ^14^, ^15^, ^16^ Therefore, systematic studies on a series of compounds from a given family are essential. In particular, a series of complexes with fine control of only one structural characteristic should be ideal to see its influence on their dynamic properties. To this point, only two large families of Dy‐based SMMs with Δ/*k*_B_ >1000 K are known: the pentagonal bipyramidal [Dy(Solv)_5_(L^1^)(L^2^)] (Solv=THF, Py; L^1^=O*t*Bu, Cl, Br, PhO; L^2^=O*t*Bu, Cl, Br, PhO) complexes^6^ and the metallocene [Dy(Cp^R1^)(Cp^R2^)]^+^ cations (R^1^=H, Me, *t*Bu, *i*Pr; R^2^=H, Me, *t*Bu, *i*Pr).^7^, ^8^, ^9^, ^10^

Here we present a family of five‐coordinate Dy SMMs, [Dy(Mes*O)_2_(THF)_2_X] (Mes*=2,4,6‐tri‐tert‐butylphenyl) (X=Cl, **1**; Br, **2**; I, **3**), designed such that the Dy coordination sphere can be selectively varied at a single position to study its influence on the relaxation dynamics. In this system, the sterically demanding aryloxide ligand was employed to reduce the coordination number at the Dy centre. Alkoxide and aryloxide‐based ligands have been widely used in Ln chemistry,^17^ and in synthesising Ln SMMs in recent years.^6^, ^14^, ^18^, ^19^, ^20^ The [Dy(Mes*O)_2_(THF)_2_X] complexes were prepared directly by the salt metathesis reactions of two equivalents of NaOMes* with the parent halide in THF (Scheme 1). Similarly, the diamagnetic Y(III) analogous compound, **1‐Y** and a 5 % doped sample **5 %Dy@1‐Y** were synthesised to perform complementary NMR spectroscopy and dilution experiments, respectively.

**Scheme 1:**
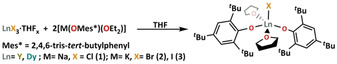
Synthesis of **1**–**3**, **1‐Y**, **2‐Y** and **3‐Y**.

The single‐crystal X‐ray analysis of **1**–**3** and **1‐Y** (Figure 1 and Figures S1–S3 in Supporting Information) reveal that **1**, **2** and **1‐Y** crystallise in the *P*2_1_/*c* space group, whereas **3** crystallises in *C*_2_/*c* (Tables S1–S2). All molecules contain a Ln^III^ ion in a rare pentacoordinate distorted square‐based pyramidal geometry, having a halide anion at the apex of the pyramid with two *trans*‐Mes*O ligands, and two *trans*‐THF molecules making the square base (Tables S3–S4). The only monomeric five‐coordinate Dy SMM known in the literature, [Dy(NHAr)_3_(THF)_2_] (Ar=C_6_H_3_ *i*Pr_2_‐2,6),^21^ has an energy barrier of 34 K, arising from a trigonal bipyramidal geometry with three anionic anilide donors and two neutral THF donors. The distortion of the coordination sphere for five‐coordinate systems can be quantified by the geometric parameter $\tau_{5}$ =(*β*−*α*)/60, where *β* and *α* are the largest and second‐largest angles in the coordination sphere, respectively. The $\tau_{5}$ parameter quantifies the degree of trigonality within the structural continuum between square‐based pyramid ($\tau_{5}$ =0) and trigonal bipyramid ($\tau_{5}$ =1).^22^ For complexes **1** to **3**, $\tau_{5}$ was found to be 0.348, 0.344 and 0.340, respectively, which is consistent with a significantly distorted square‐based pyramid (Table S4). This distortion is mainly due to the four O‐donors in the square base, which are not in a single plane. In **1**, the angle at Dy between the O‐donors from the THF ligand is 167.3(1)°, and the angle between the O‐donors from aryloxide is 146.4(1)°. Continuous shape measurement calculations also favour a square‐based pyramid (Table S5).^23^

**Figure 1:**
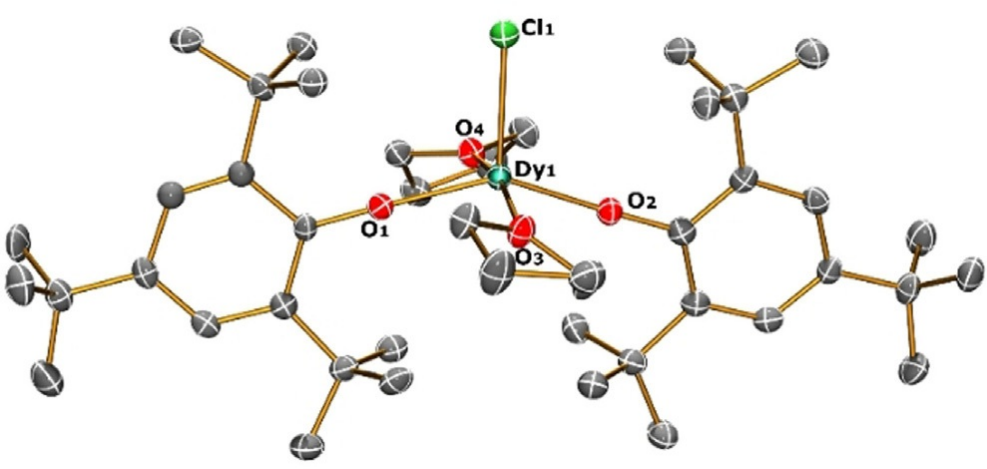
View of the [Dy(OMes*)_2_(THF)_2_Cl] molecular structure in **1** from its X‐ray crystal structure at 100 K with thermal ellipsoids drawn at 50 % probability level (Dy turquoise, Cl green, O red, C grey). H atoms are omitted for clarity.

The Dy−O bond distances for **1** to **3** (Table S3) for the anionic oxides O1 and O2 fall in the range 2.116(4) to 2.124(3) Å, whilst the neutral oxygen donors O3 and O4 show longer Dy−O bonds: for **1**, 2.370(3) and 2.352(3) Å; for **2**, 2.346(4) and 2.370(4) Å; for **3**, 2.366(3) and 2.366(3) Å. When moving from X=Cl to Br to I in the series, the Dy−X bond distance increases from 2.537(1) to 2.6981(6) to 2.9540(6) Å, which is attributed to the increase in the ionic radii of the halide present. The intermolecular nearest Dy⋅⋅Dy distances in the crystal packing (Figures S4–S12) varies as 7.7, 7.9 and 10.7 Å for **1**, **2** and **3** respectively. Complex **1‐Y** is isostructural to **1** (Tables S2–S4) with Y−Cl, Y−O1, Y−O2, Y−O3, Y−O4 distances and ∠(O1‐Y‐O2) angle being 2.529(2), 2.115(4), 2.111(4), 2.358(5), 2.327(4) Å and 145.1(2)°, respectively.

Ab initio complete active space self‐consistent field spin‐orbit (CASSCF‐SO) calculations were performed on the crystal structures of **1**–**3** to gain further insight into their electronic structures. The calculations confirm that the *trans*‐Mes*O ligand pair dominates the electronic structure and bestows strong magneto‐crystalline anisotropy on the Dy^III^ ions; the calculated magnetic axis passes through the *trans*‐Mes*O ligand pair and perpendicular to the halide (Figures S24–S26). The highest magnetic ground state *m_J_*=±15/2 (>97 %; Table S6) of the ^6^H_15/2_ multiplet is stabilised by the axial ligand field, where the energies of the first two excited states are for **1**: 596.1 and 997.7 K, for **2**: 630.6 and 1040.0 K and for **3**: 644.7 and 1063.4 K; the first excited state is well‐described as *m_J_*=±13/2 (>95 %), the second is dominated by *m_J_*=±11/2 (>80 %), but the third and all subsequent states are highly mixed (Tables S6–S9). Analysis of the transition probabilities by utilising the average matrix elements of the Cartesian magnetic moment operators between electronic states suggests the thermal relaxation to be most likely via the third excited state with an activation energy of 1099.1, 1150.9 and 1178.6 K for the Cl, Br and I analogues, respectively, (Figure 2 and Figure S23).

**Figure 2:**
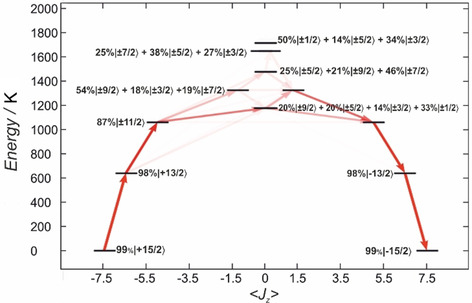
CASSCF‐SO‐calculated energy diagram of the ground‐state multiplet for **1** indicating the zero‐field magnetic transition propensities obtained from the average of the three Cartesian transition magnetic moment operators. The opacity of each arrow is proportional to the normalised transition propensity. The percentages of mixing have been rounded off to whole numbers and the contributions lower than 10 % have been omitted for clarity.

To investigate the magnetic properties of this family of complexes, dc (direct current) and ac (alternating current) susceptibility measurements were performed. The temperature dependence of the dc magnetic susceptibility was performed under an applied field of 0.1 T (Figure S28). At 270 K, the measured *χT* values of 13.8, 13.7, 13.5 and 13.7 cm^3^ mol^−1^ K, for **1**, **2**, **3** and **5 %Dy@1‐Y** (when normalised per mol of Dy complex) respectively, are close to the expected value (14.17 cm^3^ mol^−1^ K) for a free Dy^III^ ion.^24^ The continuous decrease of the *χT* product upon cooling to low temperatures suggests strong crystal‐field splitting. The field dependence of magnetisation measured at multiple temperatures below 15 K (between ±7 T) shows slow magnetisation dynamics and blocking (Figures S29–32). The magnetisation at 1.85(1) K and 7 T saturates at *M*_sat_=5.3, 5.0, 4.6 and 5.3 N_A_μ_B_ for **1**, **2**, **3** and **5 %Dy@1‐Y** (when normalised per mol of Dy complex), respectively. The slow dynamics observed by dc measurements were further studied using ac susceptibility with frequencies up to 10 kHz. As shown in Figures S33–40, the in‐phase (*χ′*) and out‐of‐phase (*χ“*) components of the ac susceptibility are strongly temperature and frequency dependent in zero‐dc field for all the complexes. The magnetisation relaxation time and its associated distribution were estimated as a function of the temperature (Figure 3) from the fitting of the experimental *χ′* versus. *ν* and *χ”* versus *ν* data to the generalised Debye model (see Figures S33–40).^25^, ^26^, ^27^

**Figure 3:**
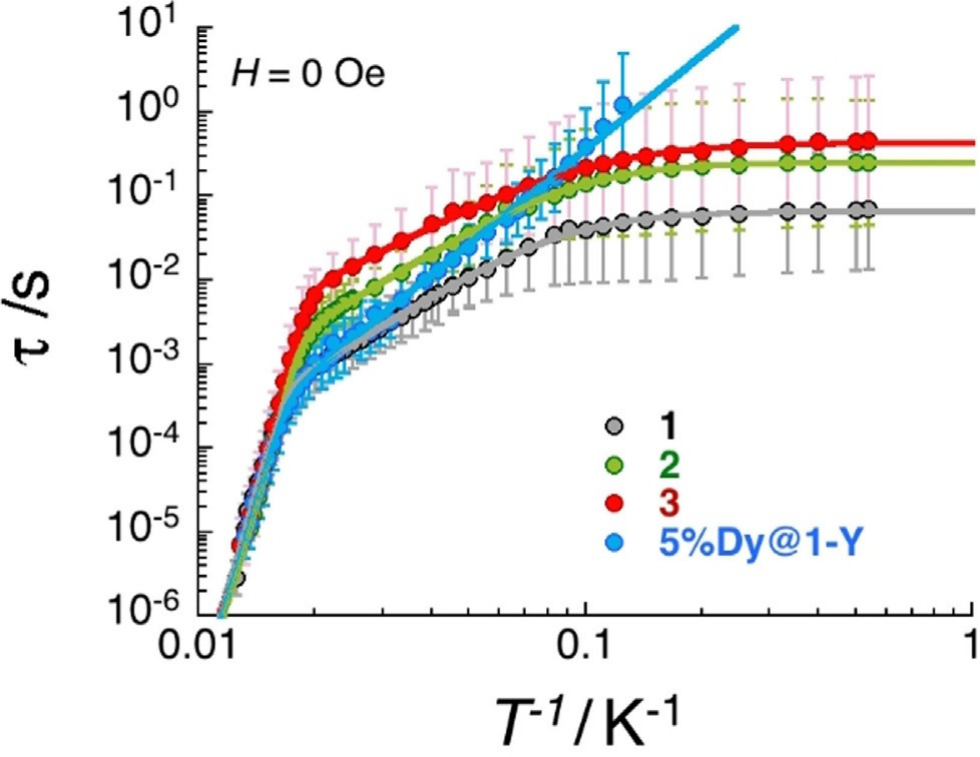
Temperature dependence of the relaxation time for **1**–**3** and **5 %Dy@1‐Y** estimated from the generalised Debye fits of the ac susceptibility data shown in Figures S33, S35, S37 and S39 collected under a zero applied field. The estimated standard deviations of the relaxation time (vertical solid bars) have been calculated from the α parameters of the generalised Debye fit (Figures S34, S36, S38 and S40) and the log‐normal distribution as described in reference 27. The solid lines are the best fit discussed in the text.

In zero dc‐field, paramagnetic relaxation^26^, ^27^, ^28^ usually involves the three main mechanisms including Raman, ^[28, 29]^ thermally activated (Orbach‐like)^28^, ^30^ and quantum tunnelling (QTM)^28^ relaxation pathways, as summarised in the following equations [Eq. (1), (2)]:

(1) $$\tau^{-1}=\tau_{Raman}^{-1}{+\;\tau }_{Orbach}^{-1}+\tau_{QTM}^{-1}$$

(2) $$\tau^{-1}=CT^{n}+\tau_{0}^{-1}exp\left(-\frac{\Delta }{k_{B}T}\right)+\tau_{QTM}^{-1}$$

As shown in Figure 3, the above five‐parameters model can reproduce almost perfectly the *τ* versus *T* ^−1^ data for **1**, **2** and **3** (see Table 1), which clearly display three temperature domains associated with dominating Orbach (above ∼55 K), Raman (∼55–10 K) and QTM (below ∼10 K) processes. It is interesting to note that the blocking temperature of these systems, *T*_B_^100s^, is not defined for **1**, **2** and **3,** as the QTM relaxation time is systematically smaller than 100 seconds. For the diluted compound, **5 %Dy@1‐Y**, the QTM regime is not observed in the available experimental window and thus the relaxation time was modelled considering only Orbach and Raman processes down to 8 K.

**Table 1:** Δ, *τ*_0_, *C*, *n* and *τ*_QTM_ parameters generated from the fit of the relaxation time‐temperature dependence (Figure 3) for **1**–**3** and **5 %Dy@1‐Y**. These parameters are given with their fitting error in parenthesis and their estimated standard deviations (±) based on the estimated standard deviations of the relaxation times shown in Figure 3.

Complex	**1**	**2**	**3**	**5 %Dy@1‐Y**
Δ/*k*_B_ [K]	1262(32)±199	1210(10)±91	1202(10)±174	1229(64)±260
*a* [*τ*_0_=10^−*a*^ s]	12.2(2)±1.2	12.1(1)±0.6	11.9(1)±1.1	12.1(4)±1.6
*c* [*C*=10^−*c*^ s^−1^ K^−*n*^]	1.9(1)±0.7	2.35(3)±0.82	2.1(1)±1.2	3.3(1)±0.4
*N*	2.9(1)±0.4	2.86(2)±0.51	2.42(4)±0.81	3.73(5)±0.25
*b* [*τ*_QTM_=10^−*b*^ s]	1.18(2)±0.25	0.59(1)±0.28	0.36(1)±0.31	≫100
*τ*_raman_^30K^ [10^−2^ s]	0.4	1.3	3.1	0.6

The first conclusion that can be drawn from these experimental results (Table 1), is that the activation energy of the Orbach process is statistically the same regardless of the halide present at Δ/*k*_B_≈1200 K, and does not change for **1** upon dilution; this result agrees with our CASSCF‐SO calculations. The lack of change in Δ/*k*_B_ is probably because the halide is not on the principal anisotropy axis. The modification of the phonon bath moving from **1** to **5 %Dy@1‐Y** also has little influence on Δ/*k*_B_. This conclusion is not surprising as the Orbach mechanism is primarily intrinsic to the electronic structure of the SMM, which is not much perturbed by the choice of halide here.

On the other hand, the halide substitution has a clear impact on the Raman relaxation with a characteristic time that increases as Cl<Br<I (Figure 3, Table 1). While the *C* and *n* parameters are relatively similar along the series, the intrinsic Raman relaxation time for a given temperature between 55 and 10 K (for example at 30 K which we define as *τ_Raman_* ^30K^) changes by a factor of 8 between **1** and **3**. In contrast to the Orbach process, dilution significantly impacts the *C* and *n* parameters which decrease (/24) and increase (×1.3), respectively. This is a striking difference to the *bis*‐cyclopentadienyl dysprosium(III) SMMs,^7^ or in some pentagonal bipyramidal Dy SMMs, where there was no difference between the Raman parameters for the pure and doped materials.6c This suggests that **1** is far more sensitive to the precise crystal lattice and associated phonon bath. It is tempting to link this sensitivity to the faster relaxation observed here.

The halide variation also varies the QTM time which increases Cl<Br<I (by a factor 6 between the Cl and I analogues; Table 1). However, this halide effect is relatively small when compared to the influence of the dilution as exemplified by at least a 20‐fold enhancement of *τ*_QTM_ in **5 %Dy@1‐Y**. The large effect of dilution on the QTM time suggests that internal dipolar fields play a significant role in the efficiency of this mechanism. Hence, to determine if the differing QTM times for **1**–**3** arise from a simple change in the effective dipolar field, or due to something more intrinsic, we have performed classical simulations of the dipolar magnetic field in each compound. Taking the crystallographic coordinates of each compound, we simulate a classical magnetic dipole *S=*1/2 with anisotropy given by the effective *g*‐values from CASSCF‐SO (Table S6), and perform stochastic spin flips under the field of all dipoles in a sphere of 40 Å radius. At the Dy^III^ ion at the centre of the sphere, we find the magnitude of the dipolar fields to be 20(7), 19(7), 15(8) and 4.8(6) Oe, for **1**, **2**, **3** and **5 %Dy@1‐Y**, respectively. Clearly the internal field for **5 %Dy@1‐Y** is far smaller than for the pure compounds, in agreement with the significantly reduced QTM. However, while there is a trend in the magnitude of the mean dipolar field for **1**–**3**, the standard deviations are significant such that these cannot be statistically distinguished. Hence, it is likely that another factor, potentially the mass or effective diffusion of charge of the halide, is also responsible for the change in the QTM time.

When considering the Dy‐based SMMs listed in Table S11 together with this new series of five‐coordinate Dy SMMs, it is relatively straightforward to draw conclusions about the key parameters that govern the different relaxation mechanisms. The first priority remains the design of the SMM to achieve the highest possible energy barrier of the Orbach relaxation. In that sense, the highest Δ/*k*_B_, 2217(15) K, is found for [Dy(C_5_ *i*Pr_5_)(C_5_Me_5_)][B(C_6_F_5_)_4_] (**6**) that displays the highest *T*_B_^100s^ (65 K).^8^ But the literature shows that is very far from the only consideration. Indeed, **6** displays also the highest *τ*_QTM_ (25 000 s) and *τ_Raman_* ^30 K^ (1195 s). Therefore, it is absolutely essential to increase simultaneously the characteristic time of the Raman and QTM processes in order to allow the Orbach mechanism to govern the magnetization relaxation over the largest possible temperature domain. The present study suggests that the use of heavier atoms within the ligand favours a significant slowing down of the Raman regime, and possibly also relaxation through QTM. Consequently, the use of more massive groups could be employed to enhance the performance further of already high‐performing SMMs.

A more impressive step in this direction is found upon dilution, which does not affect Δ/*k*_B_ but does influence both Raman and QTM processes. This could be related to the utilization of bulky counter‐anions (like [B(C_6_F_5_)_4_]^−^ or [BPh_4_]^−^) in the best performing SMMs listed in Table S11. Both dilution and the presence of large counter‐anions seem to slow the non‐Orbach processes. It is thus clear that the environment of the SMMs, needs to be controlled carefully in order to obtain high‐performing SMMs.

## Experimental

Full details of synthesis and characterisation of all materials can be found in the Supporting Information.

CCDC 1978052, 1978053, 1978054 and 1978055 contain the supplementary crystallographic data for this paper. These data are provided free of charge by The Cambridge Crystallographic Data Centre

## Conflict of interest

The authors declare no conflict of interest.

## Supporting information

As a service to our authors and readers, this journal provides supporting information supplied by the authors. Such materials are peer reviewed and may be re‐organized for online delivery, but are not copy‐edited or typeset. Technical support issues arising from supporting information (other than missing files) should be addressed to the authors.

Supplementary
