# Supplementary material for: Probing Relaxation Dynamics in Five‐Coordinate Dysprosium Single‐Molecule Magnets
Source: Chemistry. 2020 Jun 3;26(35):7774–8. doi: 10.1002/chem.202001235 (PMC7383731; doi:10.1002/chem.202001235)
Supplement: Supplementary file 1 — Supplementary [file CHEM-26-7774-s001.pdf]

Chemistry–A European Journal

Supporting Information

Probing Relaxation Dynamics in Five-Coordinate Dysprosium Single-Molecule Magnets

Vijay S. Parmar,[a] Fabrizio Ortu,[a] Xiaozhou Ma,[b] Nicholas F. Chilton,[a] Rodolphe Clérac,*[b] David P. Mills,*[a] and Richard E. P. Winpenny*[a]

Contents

1. General experimental procedures S2

2. Experimental synthesis S2

3. Crystallography S5

4. NMR spectra S20

5. IR spectra S22

6. Computational details S26

7. Magnetism S35

8. References: S50

1. General experimental procedures

All syntheses and manipulations were conducted under argon with the rigorous exclusion of oxygen and water using Schlenk line and glovebox techniques. Diethylether and hexane were dried by refluxing over potassium and were stored over potassium mirrors. All solvents were degassed before use. For NMR spectroscopy C_6D_6 was dried by refluxing over potassium. NMR solvents were vacuum transferred and degassed by three freeze-pump-thaw cycles before use. Anhydrous $LnCl_3$ (Ln = Y or Dy) were purchased from Alfa Aesar and were used as received. ^{1}H (400 MHz) and $^{13}C\{^{1}H\}$ (100 MHz and 125 MHz), NMR spectra were obtained on an Avance III 400 MHz or 500 MHz spectrometers at 298 K. These were referenced to the solvent used, or to external TMS (^{1}H, ^{13}C). ATIR spectra were recorded as microcrystalline powders using a Bruker Tensor 27 ATR-Fourier Transform infrared (ATR-FTIR) spectrometer. Elemental analyses were performed by Mrs Anne Davies and Mr Martin Jennings at The University of Manchester, UK.

2. Experimental synthesis

[Y(OMes)$_2$(THF)$_2$Cl]·3THF* ***1-Y.*** YCl_3 (0.196 g; 1 mmol) was weighed in a Schlenk that was cooled to -78 ºC before adding 20 ml of THF. The reaction mixture was refluxed with continuous stirring for one hour to obtain the $YCl_3{\cdot}(THF)_x$ adduct. This adduct was then transferred to another Schlenk containing NaOMes*·Et_2O (0.717 g; 2 mmol; see Figures S13-S14 for NMR) in 10 ml of THF. The resulting reaction mixture was stirred at room temperature for 16 hours. After the stirring was stopped, the cloudy reaction mixture settles into a white powder (NaCl) and clear, colourless supernatant (product). The colourless supernatant solution was filtered and concentrated to get colourless block-shaped crystals (0.590 g, 0.59 mmol; 59%) in about 36 hours at 0 ºC. The crystals were dried in vacuo to afford a white crystalline solid, which elemental analysis and ^{1}H NMR spectroscopy integrals indicate is partially desolvated **1-Y**, with 0.5 molecules of lattice THF remaining in the sample. Anal. Calcd for $C_{46}H_{78}ClO_{4.5}Y$: C, 66.77; H, 9.50. Found: C, 60.47; H, 9.38. Elemental analysis results show lower carbon values than predicted, which can be attributed to carbide formation from incomplete combustion. ^{1}H NMR (400.07 MHz, C_6D_6, 298 K, ppm): δ = 1.04, (m, 10H, THF), 1.41 (s, 18H, *p*-C(CH_3)$_3$), 1.74 (s, 36H, o-C(CH_3)$_3$), 3.76 (m, 10H, THF), 7.57 (s, 4H, *m*-CH) (see Figure S15(top)). $^{13}C\{^{1}H\}$ NMR (400.07 MHz, C_6D_6, 298 K, ppm): δ =

25.04 (s), 30.48 (s), 32.01 (s), 32.08 (s), 34.58 (s), 35.92 (s), 71.58, 122.10 (s), 137.74 (s), 138.09 (s) (see Figure S15(bottom)). FTIR (ATR, microcrystalline): ῦ = 2955 (m, br), 2871 (w, br), 1458 (w), 1384 (w), 1358 (w), 1240 (s), 1201 (m), 1152 (w), 1119 (m), 1046 (m), 917 (w), 836 (s, br), 779 (w), 748 (m), 673 (w), 644 (w), 534 (m), 458 (m) cm^{-1} (see Figures S8, S9, S10 and S11).

[Dy(OMes)$_2$(THF)$_2$Cl]·3THF* ***1***. The synthetic procedure and crystallization employed for **1** are identical to that of **1-Y** using $DyCl_3$ (0.269 g; 1 mmol). Complex **1** was obtained as pale yellow coloured crystals (0.424 g, 0.39 mmol; 39%). The crystals were dried in vacuo to afford a white crystalline solid, which elemental analysis and NMR analysis of its Y analogue (**1-Y**) indicate is partially desolvated **1**, with half a molecules of lattice THF remaining in the sample. Anal. Calcd for $C_{48}H_{78}ClO_{4.5}Dy$: C, 61.31; H, 8.72. Found: C, 59.43; H, 8.83. Elemental analysis results show lower carbon values than predicted, which we attribute to carbide formation from incomplete combustion. μ_{eff} = 10.38 μ_B mol^{-1}, χT = 13.47 cm^3 mol^{-1} K (Evans method).[1,2] FTIR (ATR, microcrystalline): ῦ = 2955 (m, br), 2904 (w, br), 1456 (w), 1384 (w), 1358 (w), 1231 (s), 1201 (m), 1152 (w), 1119 (m), 1011 (m), 921 (w), 832 (s, br), 781 (w), 746 (m), 673 (w), 642 (w), 530 (m), 452 (m) cm^{-1} (see Figures S16, S20, S21 and S22).

[Dy(OMes)$_2$(THF)$_2$Br]·3THF* ***2.*** A mixture of $DyBr_3$ (0.403 g; 1 mmol) and two equivalents of KOMes* (0.605 g; 2 mmol) weighed in a 100 ml Schlenk was cooled to -78ºC before adding 30ml of THF. The reaction mixture was warmed to 74ºC and refluxed with continuous stirring for 1.5 hours, then stirred at room temperature for further 16 hours. After the stirring was stopped, the cloudy reaction mixture settles into a grey powder (KBr) and clear, colourless supernatant (product). The supernatant was filtered out to a 20 mL Schlenk and concentrated further to get colourless block-shaped crystals (0.672 g, 0.59 mmol; 59%) when stored for 36 hours at 0ºC. The crystals were dried in vacuo to afford a white crystalline solid, which elemental analysis indicate is partially desolvated **2**, with one molecules of lattice THF remaining in the sample. Anal. Calcd for $C_{48}H_{82}BrO_5Dy$: C, 58.73; H, 8.42. Found: C, 58.86; H, 8.53. μ_{eff} = 10.40 μ_B mol^{-1}, χT = 13.52 cm^3 mol^{-1} K (Evans method).[1,2] FTIR (ATR, microcrystalline): ῦ = 2955 (m, br), 2904 (w, br), 1456 (w), 1384 (w), 1358 (w), 1229 (s), 1201 (m), 1152 (w),

1117 (m), 1011 (m), 921 (w), 830 (s, br), 779 (w), 744 (m), 673 (w), 642 (w), 530 (m), 452 (m) cm^{-1} (see Figures S17, S20, S21 and S22).

[Dy(OMes)$_2$(THF)$_2$I]·2THF* ***3*.** The synthetic procedure and crystallization employed for **3** are identical to that of **2** using DyI_3 (0.544 g; 1 mmol). Complex **3** was obtained as colourless block-shaped crystals (0.355 g, 0.32 mmol; 32%). The crystals were dried in vacuo to afford a plale brown crystalline solid, which elemental analysis results indicate is **3**. Anal. Calcd for $C_{52}H_{90}IO_6Dy$: C, 56.74; H, 8.24. Found: C, 56.08; H, 8.25. μ_{eff} = 10.27 μ_B mol^{-1}, χT = 13.17 cm^3 mol^{-1} K (Evans method).[1,2] FTIR (ATR, microcrystalline): ũ = 2955 (m, br), 2904 (w, br), 1415 (w), 1384 (w), 1358 (w), 1227 (s), 1199 (m), 1154 (w), 1117 (m), 1013 (m), 919 (w), 830 (s, br), 779 (w), 744 (m), 671 (w), 642 (w), 530 (m), 452 (m) cm^{-1} (see Figures S18, S20, S21 and S22).

5%Dy@[Y(OMes)$_2$(THF)$_2$Cl]* **.** The synthetic procedure and crystallization employed here are identical to that of **1-Y** using $DyCl_3$ (0.013 g; 0.05 mmol) and YCl_3 (0.186 g; 0.95 mmol). The product was obtained as colourless block-shaped crystals (0.362 g, 0.46 mmol; 46%). Anal. Calcd for $C_{44}HClO_4Dy_{0.05}Y_{0.95}$: C, 66.47; H, 9.38. Found: C, 64.70; H, 9.56. Y/Dy composition (ICP-OES): Y, 94.8; Dy, 5.2.

3. Crystallography

The crystal data for complexes **1**, **2**, **3** and **1-Y** are compiled in Table S1 and Table S2. Crystals of **1**, and **3** were examined using an Oxford Diffraction Supernova diffractometer, equipped with CCD area detector and a mirror-monochromated Mo K_α radiation (λ = 0.71073 Å). Crystals of **2** were examined using an Oxford Diffraction Xcalibur diffractometer, equipped with CCD area detector and a mirror-monochromated Mo Kα radiation (λ = 0.71073 Å). Crystals of **1-Y** were examined using a Bruker Apex II diffractometer, equipped with CCD area detector and a graphite-monochromated Cu Kα radiation (λ = 1.54178 Å). Intensities were integrated from data recorded on 1° frames by ω rotation. Cell parameters were refined from the observed positions of all strong reflections in each data set. A multi-scan absorption correction with a beam profile was applied.[3] The structures were solved using direct methods by SHELXS; the datasets were refined by full-matrix least-squares on all unique F^2 values [4] CrysAlisPro [3] was used for control and integration, and SHELX[4,5] was employed through OLEX2[6] for structure solution and refinement. ORTEP-3[7] and POV-Ray[8] were employed for molecular graphics. CCDC 1978052-1978055 contain the supplementary crystal data for this article. These data can be obtained free of charge from the Cambridge Crystallographic Data Centre via www.ccdc.cam.ac.uk/data_request/cif. Single crystals of **5%Dy@1-Y** were screened in order to verify the doped sample was of the correct structure; these afforded cell parameters consistent with those of **1** and **1-Y**: a = 17.5646(8) Å, b = 15.9911(8) Å, c = 20.6806(7) Å, α = 90°, β = 102.270(4)°, γ = 90°, V = 5676.0(4) $Å^3$.

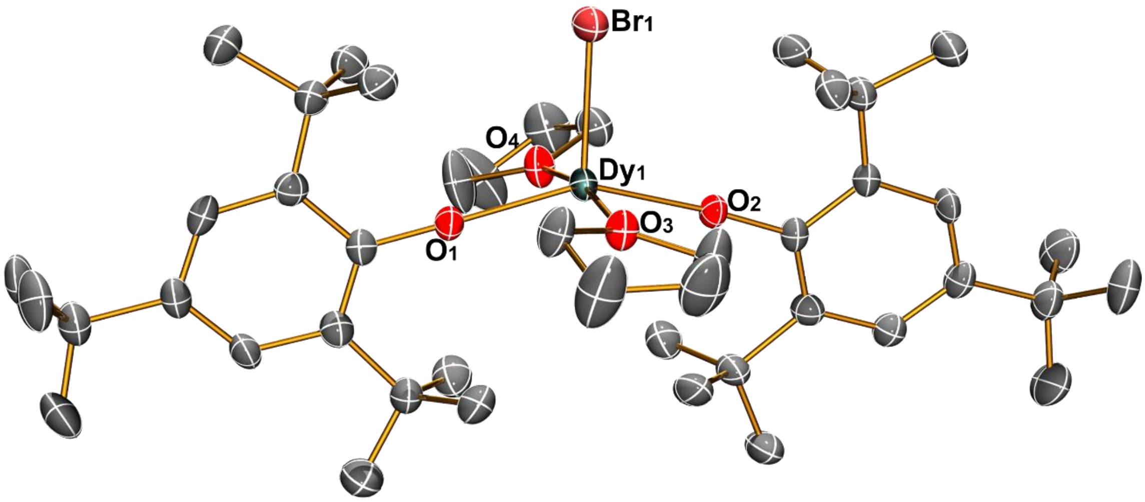

Figure S1: View of the [Dy(OMes*)$_2$(THF)$_2$Br] molecular structure in **2** from its X-ray crystal structure at 150 K with thermal ellipsoids drawn at 50% probability level (Dy turquoise, Br gold, O red, C grey). H atoms and lattice solvent are omitted for clarity.

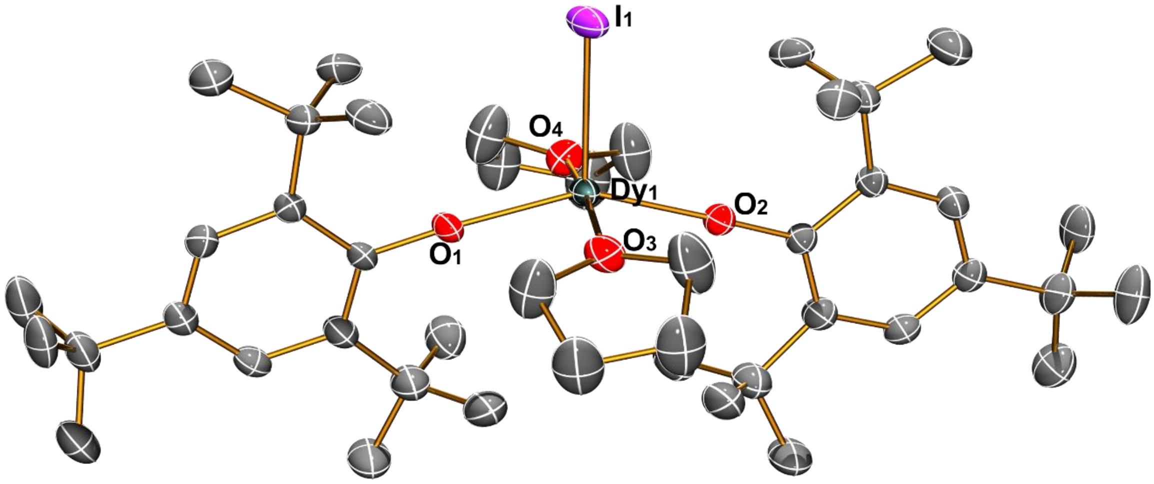

Figure S2: View of the [Dy(OMes*)$_2$(THF)$_2$I] molecular structure in **3** from its X-ray crystal structure at 150 K with thermal ellipsoids drawn at 50% probability level (Dy turquoise, Y purple, O red, C grey). H and lattice solvent atoms are omitted for clarity.

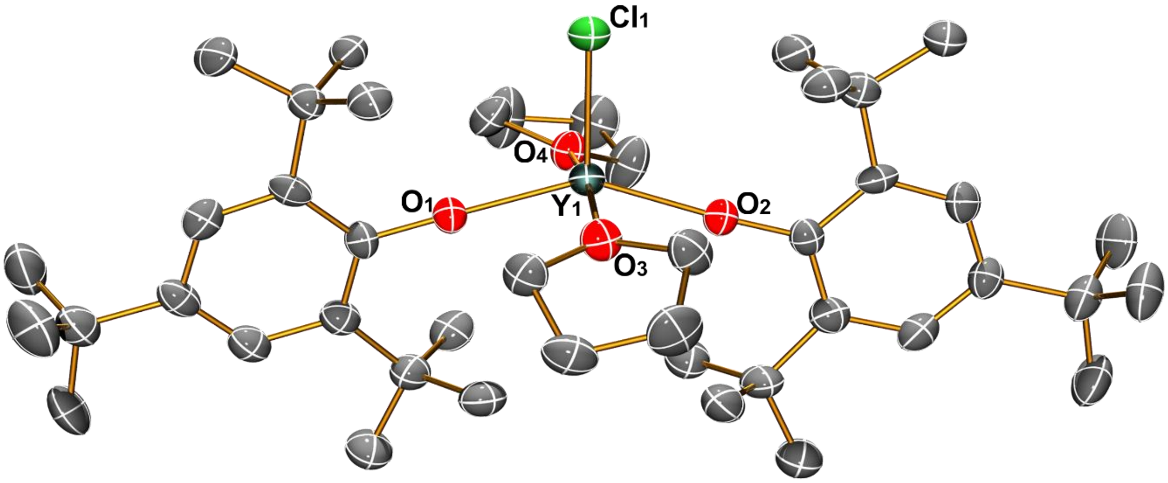

Figure S3: View of the [Y(OMes*)$_2$(THF)$_2$Cl] molecular structure in **1-Y** from its X-ray crystal structure at 150 K with thermal ellipsoids drawn at 50% probability level (Dy turquoise, Cl green, O red, C grey). H atoms and lattice solvent are omitted for clarity.

Table S1. Crystallographic data for **1**, **2** and **3.**

	**1**	**2**	**3**
Formula	$C_{56}H_{98}ClDyO_7$	$C_{56}H_{98}BrDyO_7$	$C_{52}H_{90}IDyO_6$
Fw	1081.33	1125.75	1100.63
Crystal size, mm	0.214 × 0.231 × 0.25	0.22 × 0.29 × 0.48	0.07 × 0.09 × 0.12
Temperature, K	100	150	150
Crystal system	monoclinic	monoclinic	monoclinic
Space group	$P2_1/c$	$P2_1/c$	$C2/c$
a, Å	18.2032(5)	17.6562(4)	18.7399(6)
b, Å	15.1042(5)	16.0269(3)	10.5463(4)
c, Å	20.7545(7)	20.7311(4)	27.7967(8)
α, °	90°	90°	90
β, °	99.431(3)°	102.230(2)°	94.770(3)
γ, °	90°	90°	90
V, $Å^3$	5629.2(3)	5733.2(2)	5474.6(3)
Z	4	4	4
ρ_{calcd}, g cm^3	1.276	1.304	1.335
μ, mm^{-1}	1.422	2.046	1.971
F(*000*)	2292	2364	2276
nb. of reflections (unique)	32566 (11493)	59086 (11714)	21465(6517)
nb. of parameters	576	696	377
S^a	1.082	1.041	1.017
R_1 ($I > 2\sigma(I)$)	0.0504	0.0505	0.0449
wR_2 *all*	0.1242	0.1240	0.1138
R_{int}	0.0497	0.0698	0.0557
min./max. diff map, $Å^{-3}$	–1.145, 3.352	–0.998, 2.028	–1.367, 1.312

[a]Conventional $R = \Sigma||F_o| - |F_c||/\Sigma|F_o|$; $R_w = [\Sigma w(F_o^2 - F_c^2)^2/\Sigma w(F_o^2)^2]^{1/2}$; $S = [\Sigma w(F_o^2 - F_c^2)^2/\text{no. data} - \text{no. params})]^{1/2}$ for all data.

Table S2. Crystallographic data for **1** and **1-Y.**

	**1**	**1-Y**
Formula	$C_{56}H_{98}ClDyO_7$	$C_{56}H_{98}ClO_7Y$
Fw	1081.33	1007.70
Crystal size, mm	0.214 × 0.231 × 0.25	0.1 × 0.3 × 0.3
Temperature / K	100	150
Crystal system	monoclinic	monoclinic
Space group	$P2_1/c$	$P2_1/c$
a, Å	18.2032(5)	18.2094(5)
b, Å	15.1042(5)	15.2650(3)
c, Å	20.7545(7)	20.7616(5)
α, °	90°	90
β, °	99.431(3)°	99.492(3)
γ, °	90°	90
V, Å^3	5629.2(3)	5692.0(2)
Z	4	4
ρ_{calcd}, g cm^3	1.276	1.176
μ, mm^{-1}	1.422	2.232
F(*000*)	2292	2184
nb. of reflections (unique)	32566 (11493)	15778(15778)
nb. of parameters	576	661
S^a	1.083	1.026
R_1 ($I > 2\sigma(I)$)	0.0504	0.0807
wR_2	0.1242	0.2119
R_{int}	0.0497	0.2704
min./max. diff map, Å^{-3}	–1.145, 3.352	–0.670, 1.020

[a]Conventional $R = \Sigma||F_o| - |F_c||/\Sigma|F_o|$; $R_w = [\Sigma w(F_o^2 - F_c^2)^2/\Sigma w(F_o^2)^2]^{1/2}$; $S = [\Sigma w(F_o^2 - F_c^2)^2/\text{no. data} - \text{no. params})]^{1/2}$ for all data.

Table S3: Selected bond lengths (Å) in complexes **1**, **2**, **3** and **1-Y.**

	**1**	**2**	**3**	**1-Y**
Ln-O1	2.124(3)	2.116(3)	2.124(3)	2.115(4)
Ln-O2	2.121(3)	2.120(3)	2.124(3)	2.111(4)
Ln-O3	2.370(3)	2.346(4)	2.366(3)	2.358(5)
Ln-O4	2.352(3)	2.370(4)	2.366(3)	2.327(4)
Ln-X	2.537(1)	2.6981(6)	2.9540(6)	2.529(2)

Table S4: Selected bond angles (°) in complexes **1**, **2**, **3** and **1-Y.**

	**1**	**2**	**3**	**1-Y**
O1-Ln-O2	146.4(1)	148.3(1)	148.3(2)	145.1(2)
O1-Ln-O3	86.6(1)	88.3(1)	88.0(1)	87.1(2)
O1-Ln-O4	91.8(1)	86.1(1)	88.0(1)	90.7(2)
O2-Ln-O3	85.8(1)	90.9(1)	88.9(1)	86.1(2)
O2-Ln-O4	88.6(1)	88.9(1)	88.9(1)	88.8(2)
O3-Ln-O4	167.3(1)	168.9(1)	168.8(1)	167.8(2)
X-Ln-O1	108.82(9)	108.0(1)	105.85(9)	109.3(1)
X-Ln-O2	104.35(9)	103.4(1)	105.85(9)	105.5(1)
X-Ln-O3	94.53(8)	98.43(9)	95.61(8)	95.0(1)
X-Ln-O4	97.95(8)	92.4(1)	95.61(8)	97.0(1)

Table S5: Continuous Shape Measures (**CShM**)[9] calculations for complexes **1-3**.

	**Structure**[a]	PP-5	vOC-5	TBPY-5	SPY-5	JTBPY-5
**1**	**CShM**	33.641	2.700	2.599	1.012	4.550
**2**	**CShM**	32.722	3.075	2.729	1.194	5.028
**3**	**CShM**	35.235	4.035	3.092	1.539	5.967

[a] PP-5 Pentagon (D_{5h})

vOC-5 Vacant octahedron (C_{4v})

TBPY-5 Trigonal bipyramid (D_{3h})

SPY-5 Spherical square pyramid (C_{4v})

JTBPY-5 Johnson trigonal bipyramid J12 (D_{3h})

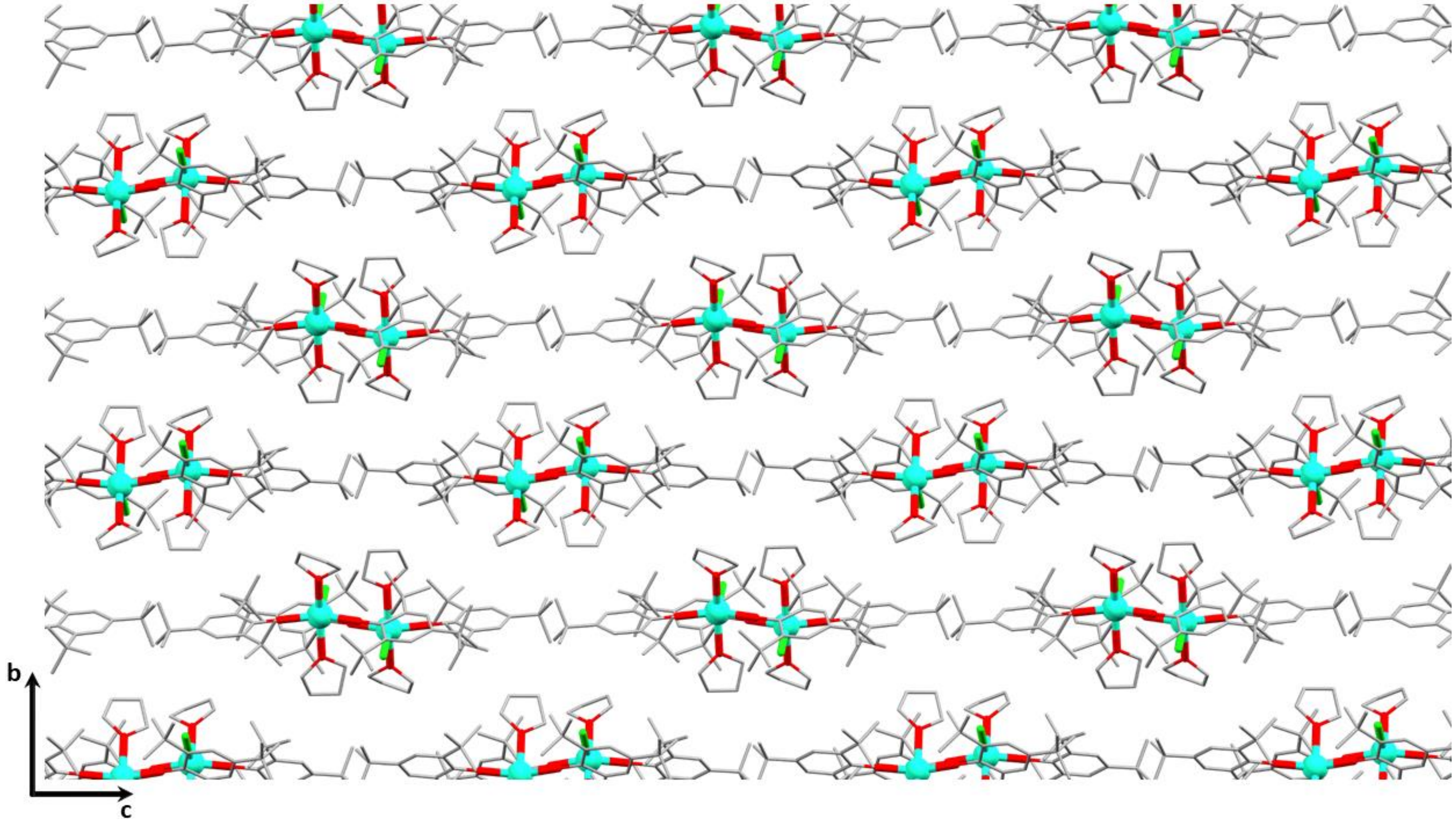

Figure S4: View of the crystal packing in [Dy(OMes*)$_2$(THF)$_2$Cl] **1** along the x-axis (Dy turquoise, I purple, O red, C grey). H atoms and lattice solvent are omitted for clarity.

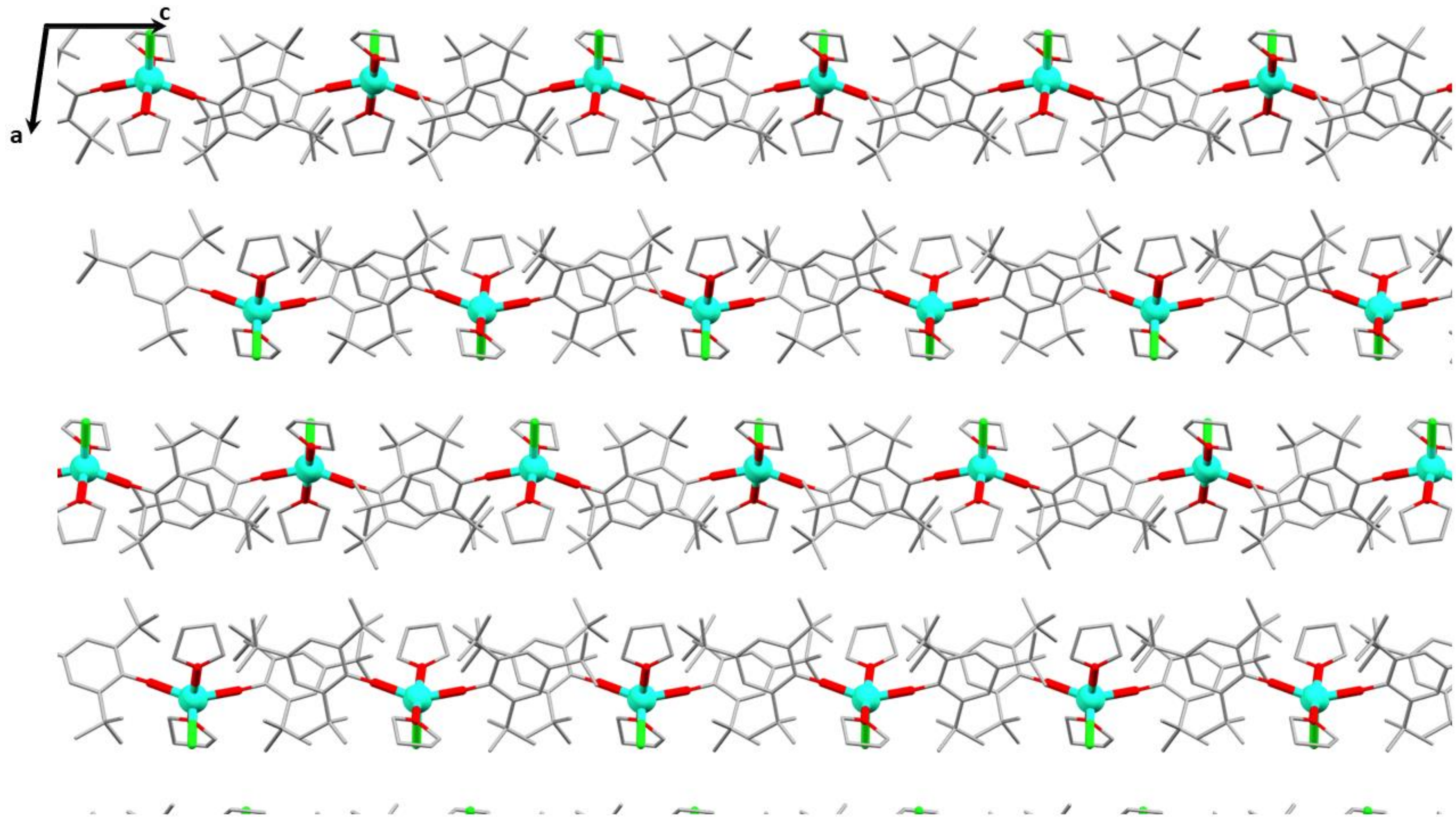

Figure S5: View of the crystal packing in [Dy(OMes*)$_2$(THF)$_2$Cl] **1** along the y-axis (Dy turquoise, I purple, O red, C grey). H atoms and lattice solvent are omitted for clarity.

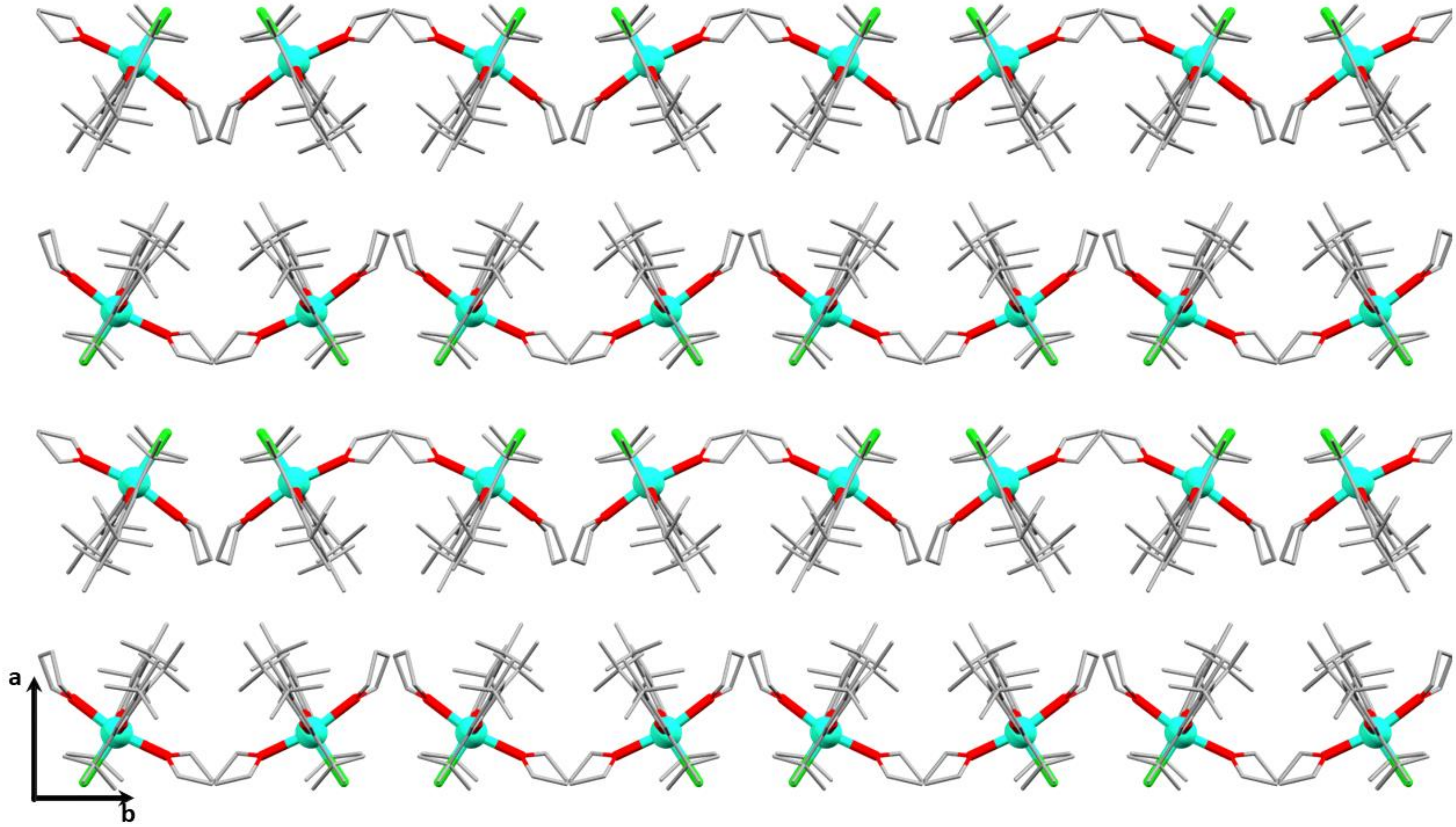

Figure S6: View of the crystal packing in [Dy(OMes*)$_2$(THF)$_2$Cl] **1** along the z-axis (Dy turquoise, I purple, O red, C grey). H atoms and lattice solvent are omitted for clarity.

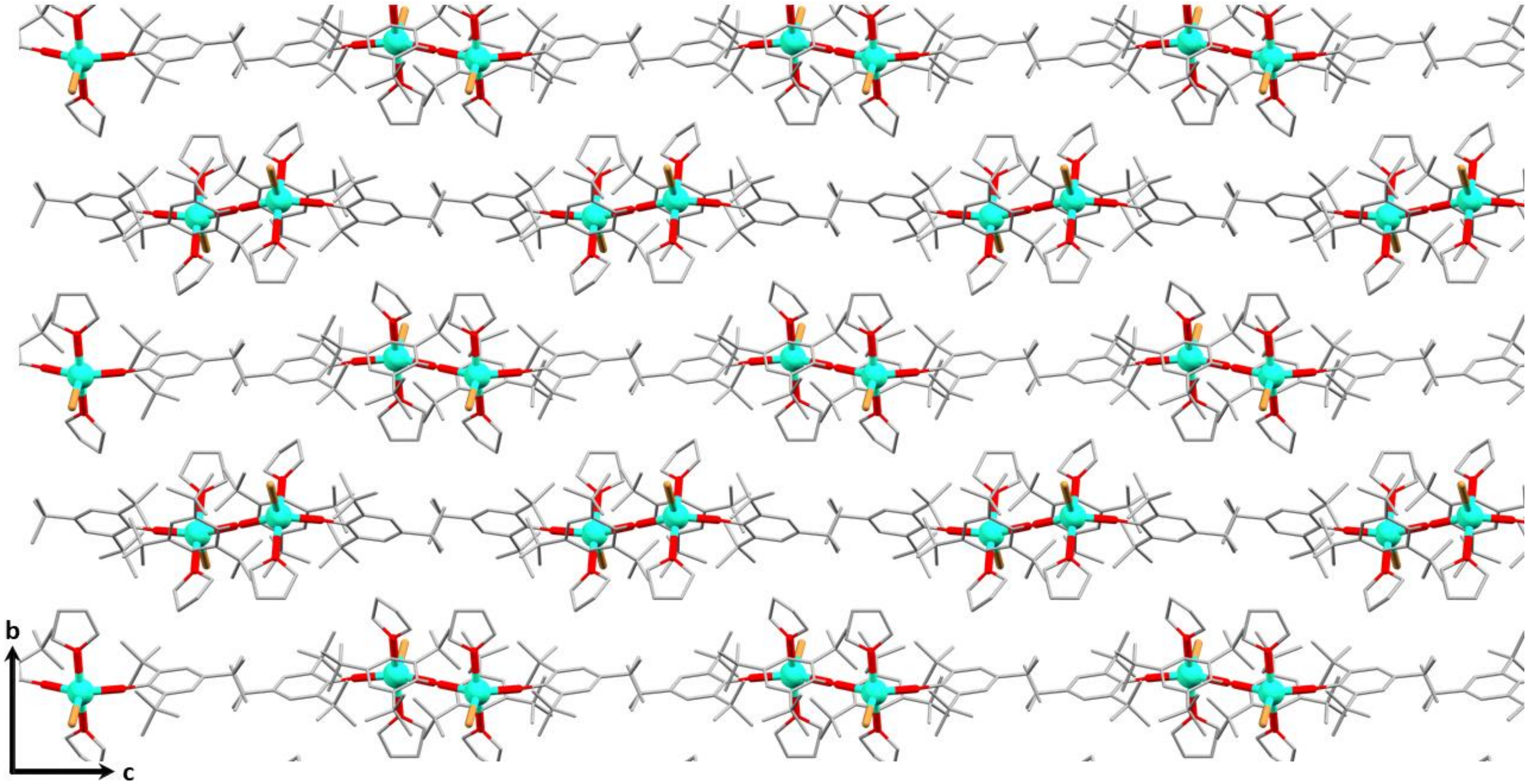

Figure S7: View of the crystal packing in [Dy(OMes*)$_2$(THF)$_2$Br] **2** along the x-axis (Dy turquoise, I purple, O red, C grey). H atoms and lattice solvent are omitted for clarity.

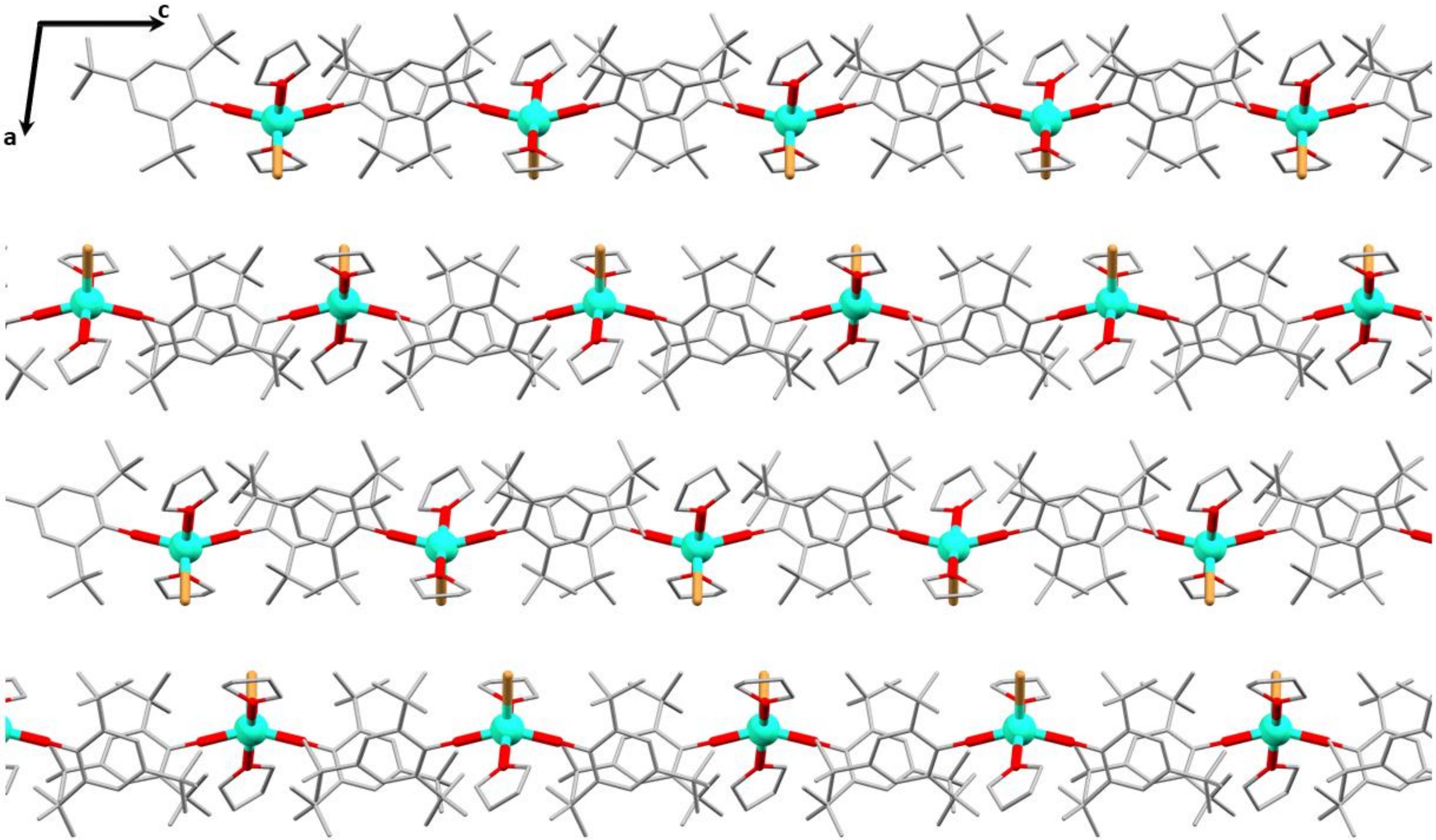

Figure S8: View of the crystal packing in [Dy(OMes*)$_2$(THF)$_2$Br] **2** along the y-axis (Dy turquoise, I purple, O red, C grey). H atoms and lattice solvent are omitted for clarity.

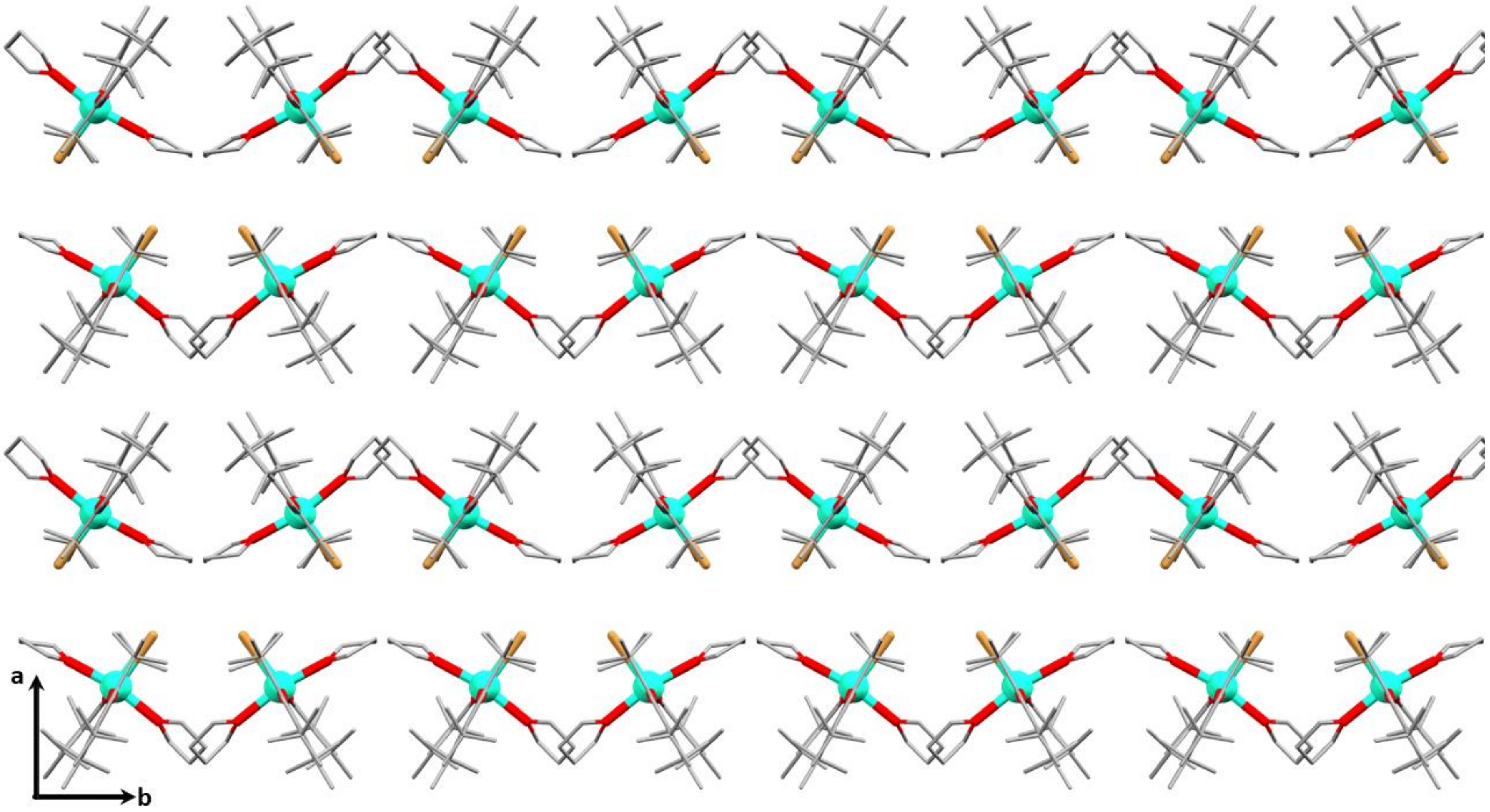

Figure S9: View of the crystal packing in [Dy(OMes*)$_2$(THF)$_2$Br] **2** along the z-axis (Dy turquoise, I purple, O red, C grey). H atoms and lattice solvent are omitted for clarity.

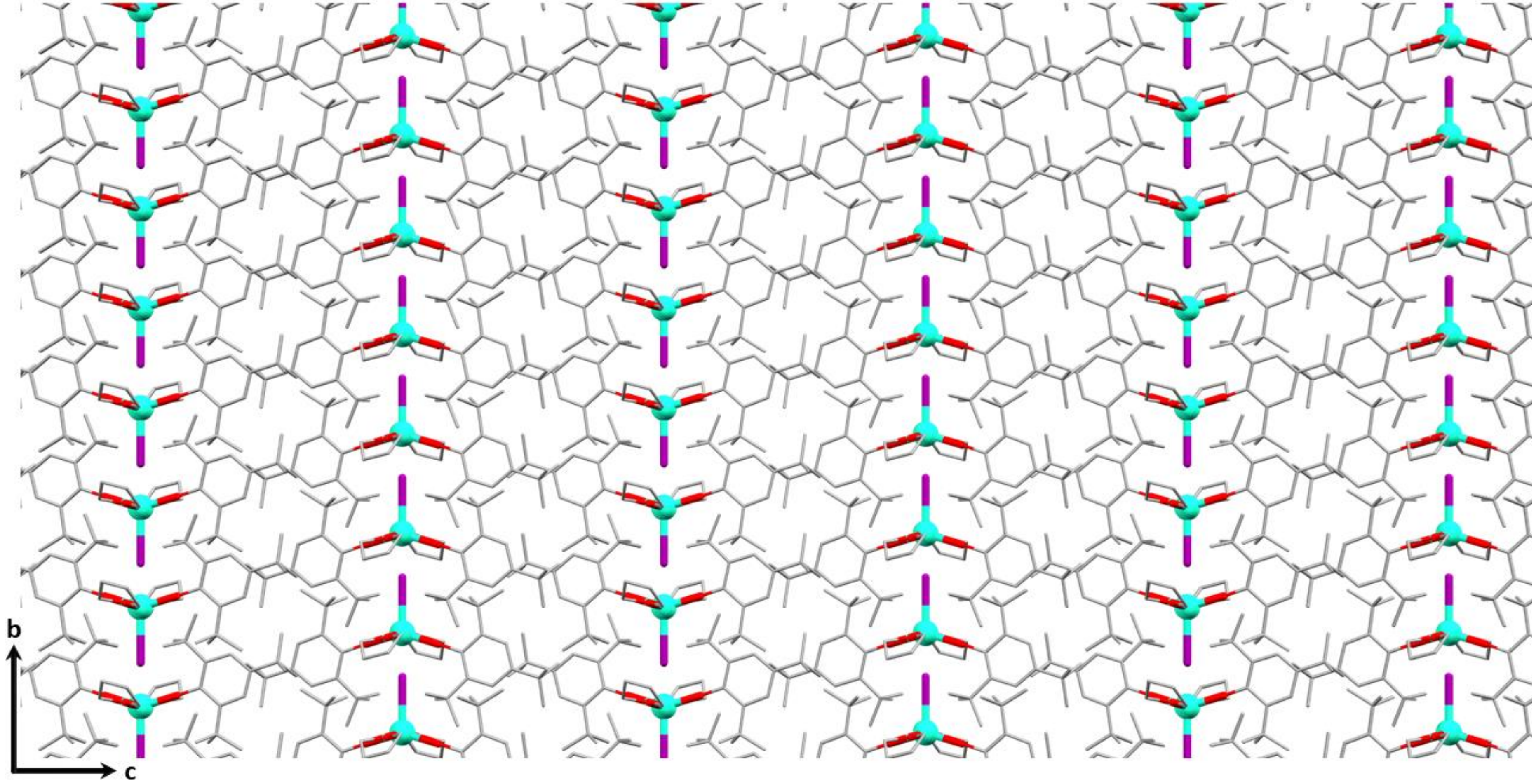

Figure S10: View of the crystal packing in [Dy(OMes*)$_2$(THF)$_2$I] **3** along the x-axis (Dy turquoise, I purple, O red, C grey). H atoms and lattice solvent are omitted for clarity.

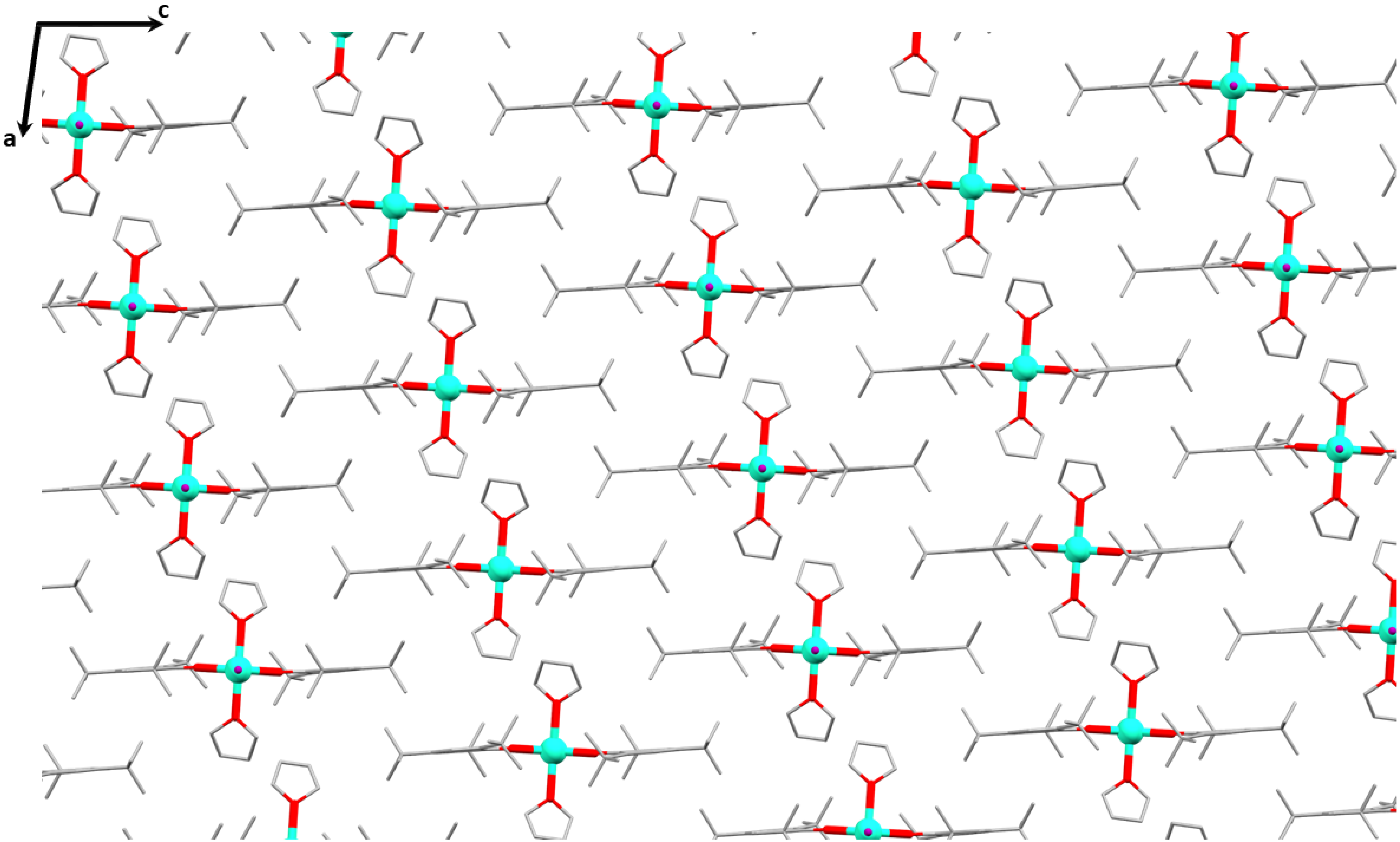

Figure S11: View of the crystal packing in [Dy(OMes*)$_2$(THF)$_2$I] **3** along the y-axis (Dy turquoise, I purple, O red, C grey). H atoms and lattice solvent are omitted for clarity.

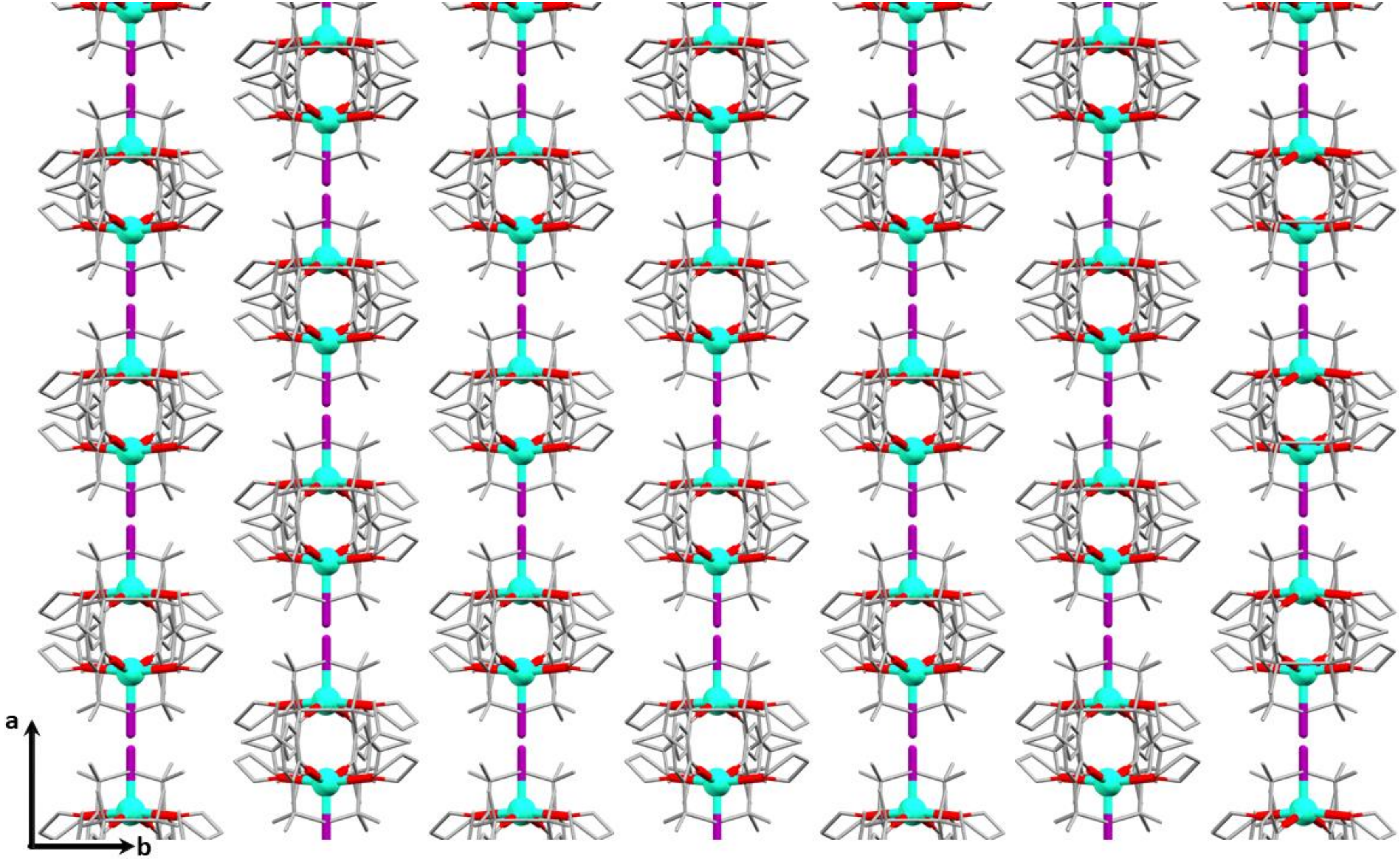

Figure S12: View of the crystal packing in [Dy(OMes*)$_2$(THF)$_2$I] **3** along the z-axis (Dy turquoise, I purple, O red, C grey). H atoms and lattice solvent are omitted for clarity.

4. NMR spectra

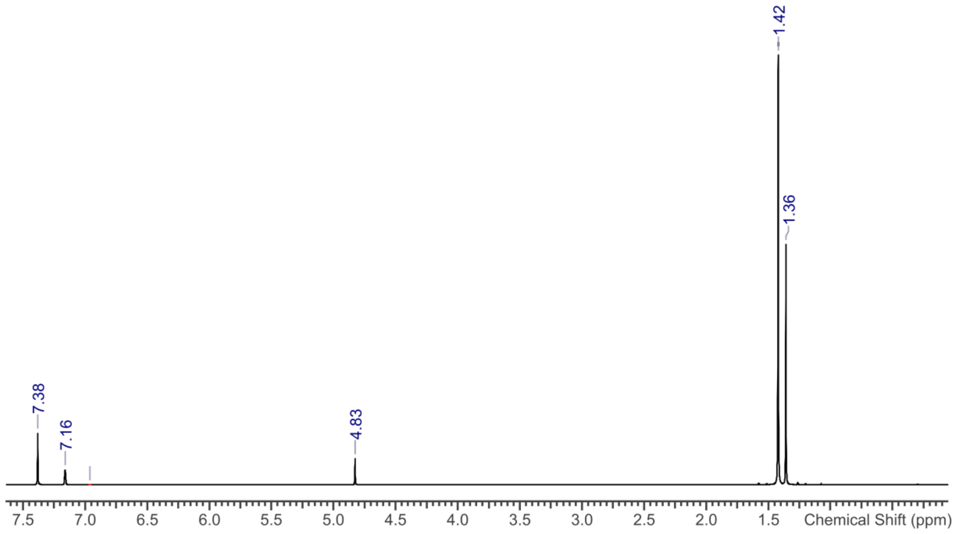

Figure S13. ^1H NMR spectrum of ligand precursor Mes*OH (2,4,6-tri-*tert*-butylphenol) in benzene (solvent reference peak: 7.16).

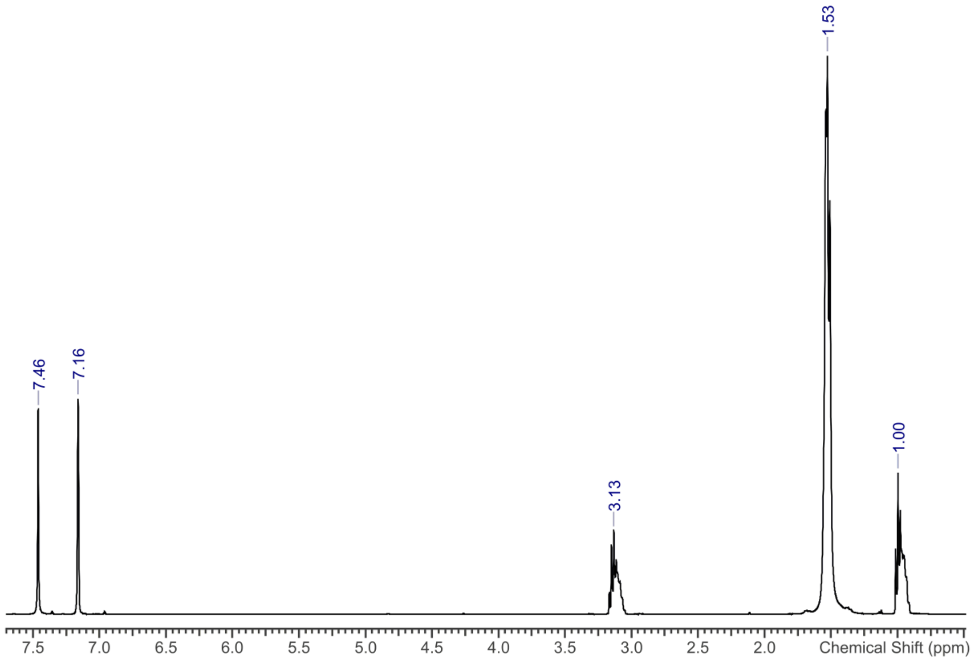

Figure S14. ^1H NMR spectrum of group 1 transfer agent Mes*ONa·Et_2O in benzene (solvent reference peak: 7.16).

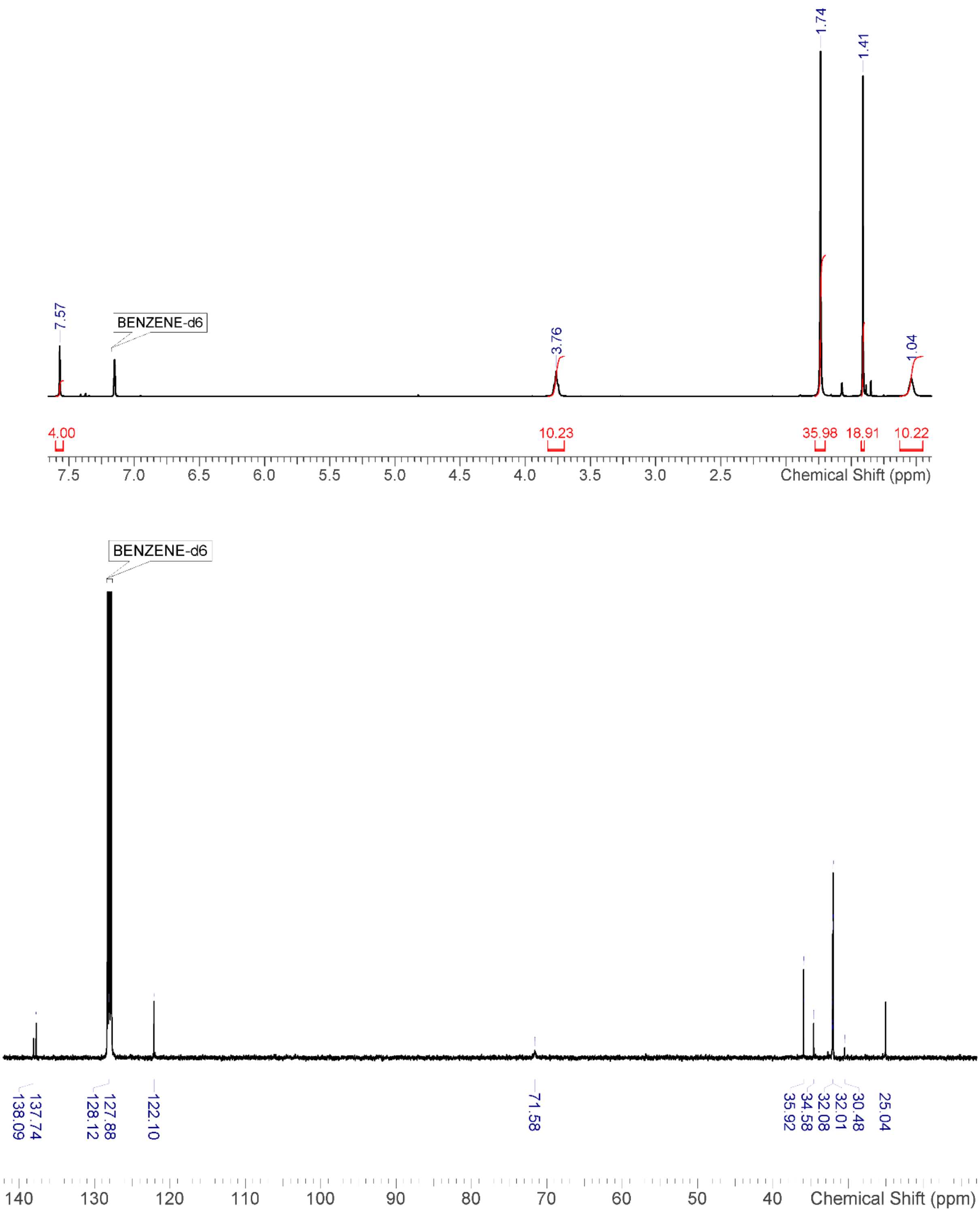

Figure S15. ^{1}H NMR (top) and ^{13}C NMR (bottom) spectrum of **1-Y** $[Y(OMes^*)_2(THF)_2Cl]$ in benzene-d_6. For ^{1}H NMR, integrals at 3.76 and 1.04 show partially desolvated compound.

5. IR spectra

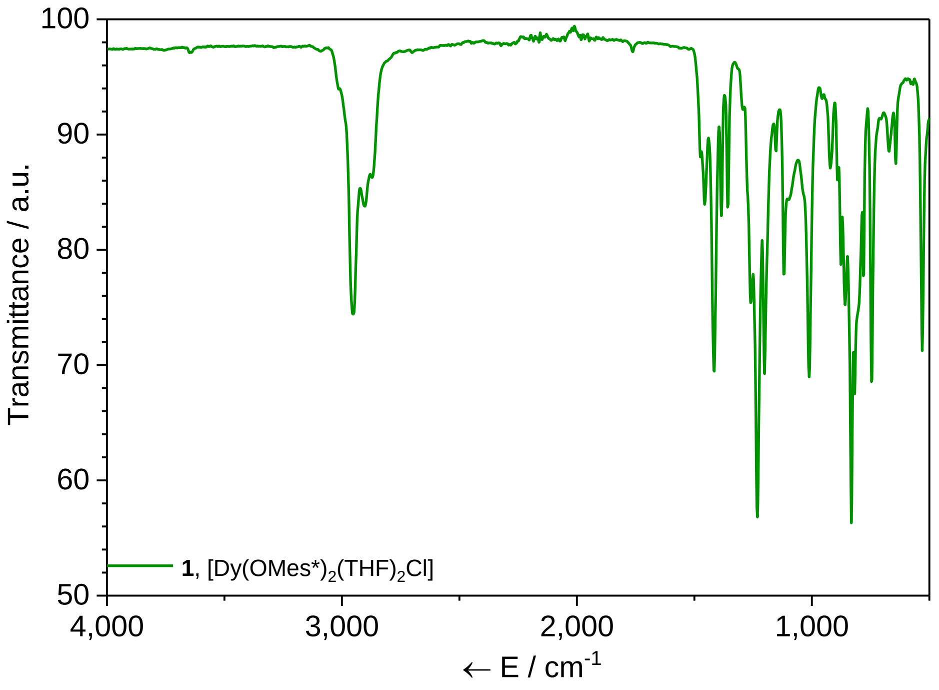

Figure S16. ATIR spectrum of **1**, 394-4000 cm^{-1}.

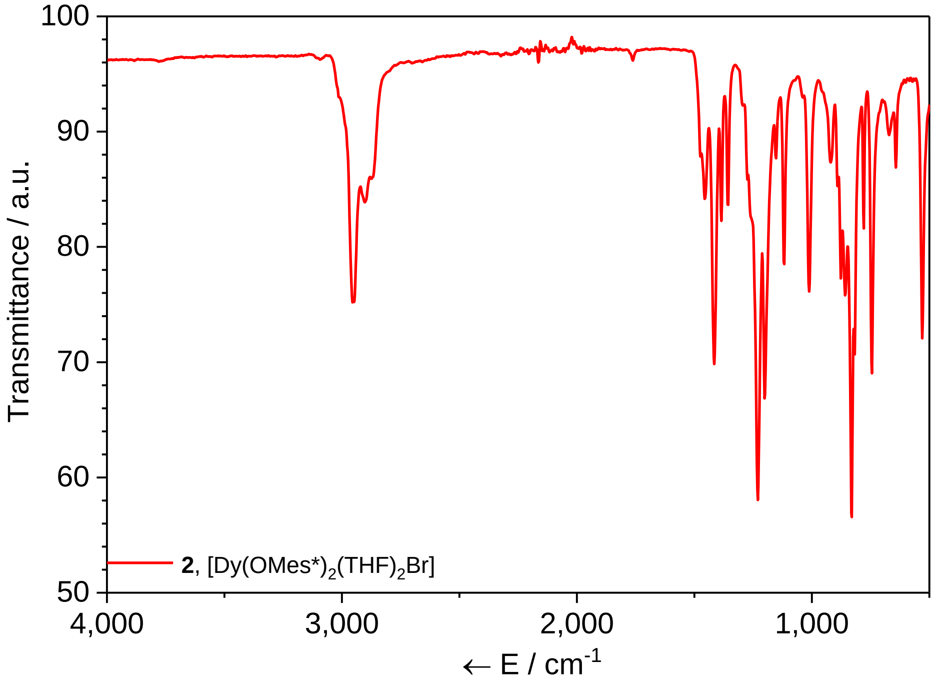

Figure S17. ATIR spectrum of **2**, 394-4000 cm^{-1}.

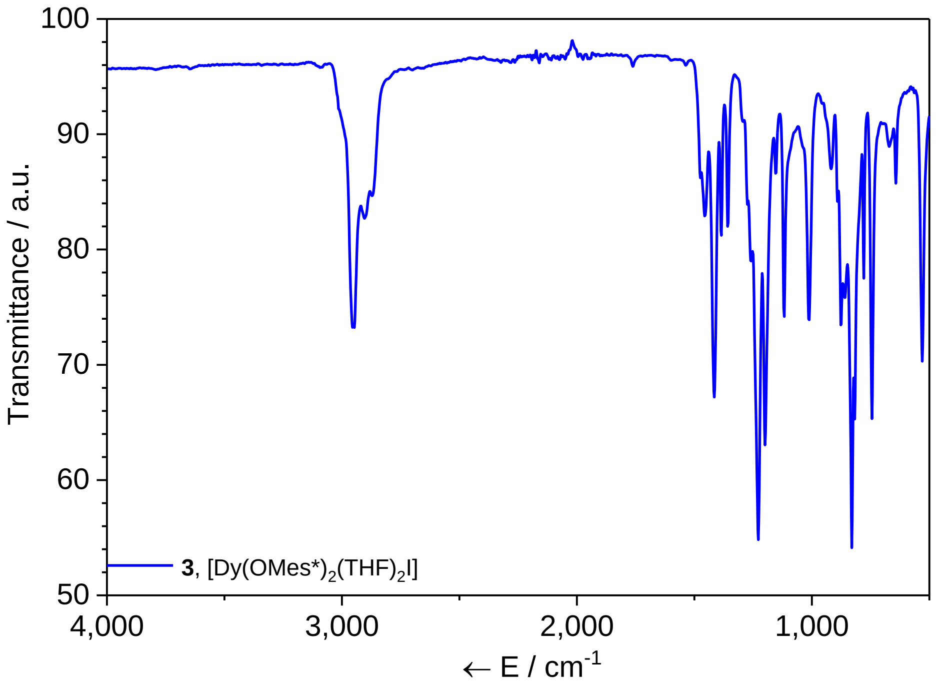

Figure S18. ATIR spectrum of **3**, 394-4000 cm^{-1}

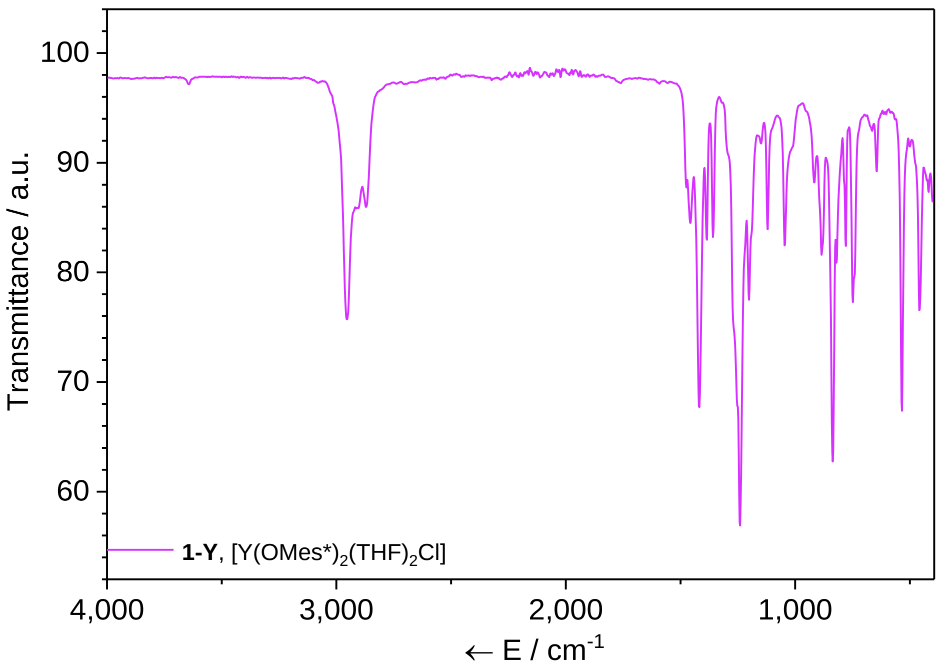

Figure S19. ATIR spectrum of **1-Y**, 394-4000 cm^{-1}.

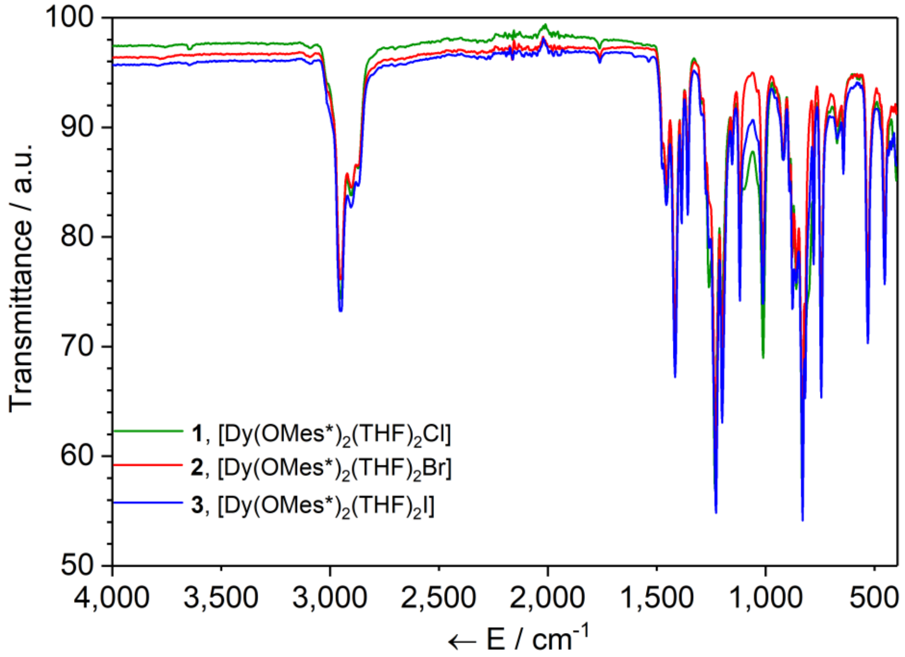

Figure S20. ATIR spectrum of **1-3**, 394-4000 cm^{-1}.

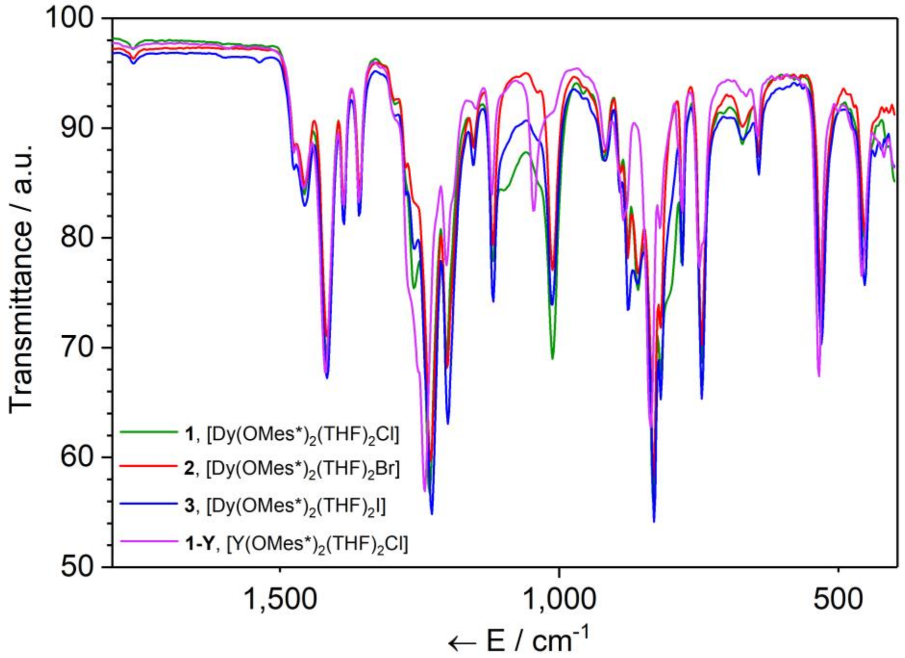

Figure S21. ATIR spectrum of **1-3** and **1-Y**, 394-1800 cm^{-1}.

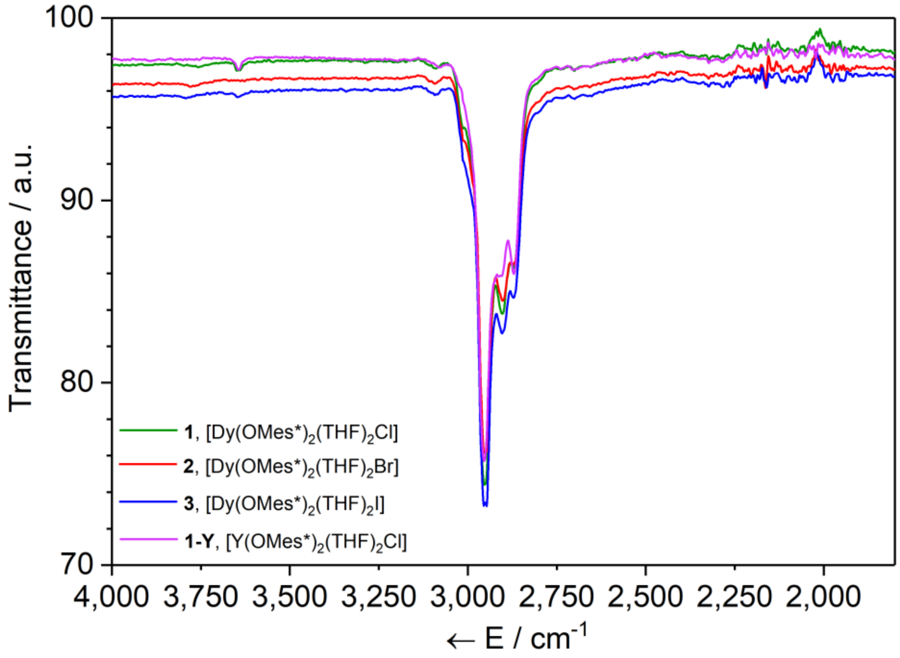

Figure S22. ATIR spectrum of **1-3** and **1-Y**, 1800-4000 cm^{-1}.

6. Computational details

All the first-principle complete active space self-consistent field spin-orbit (CASSCF-SO) calculations have been performed with MOLCAS 8.0[10] software package. The coordinates obtained from single-crystal X-ray structure were used without optimisation. In the active space, we have performed state-average CASSCF calculations with nine electrons in the 7 4f orbitals (i.e. CAS(9,7)) for 21, 224 and 490 roots for the sextet, quartet and doublet spin states, respectively. The ANO-RCC basis set library[11,12] has been used for all the atoms ([ANO-RCC-VTZP] for Dy, [ANO-RCC-VDZP] for O, and [ANO-RCC-VDZ] for C and H) with scalar relativistic effects accounted using the second-order Douglas–Kroll–Hess transformation. The crystal field decomposition of the ground J = 15/2 multiplet of the ^{6}H term was performed using the SINGLE_ANISO[13] module.

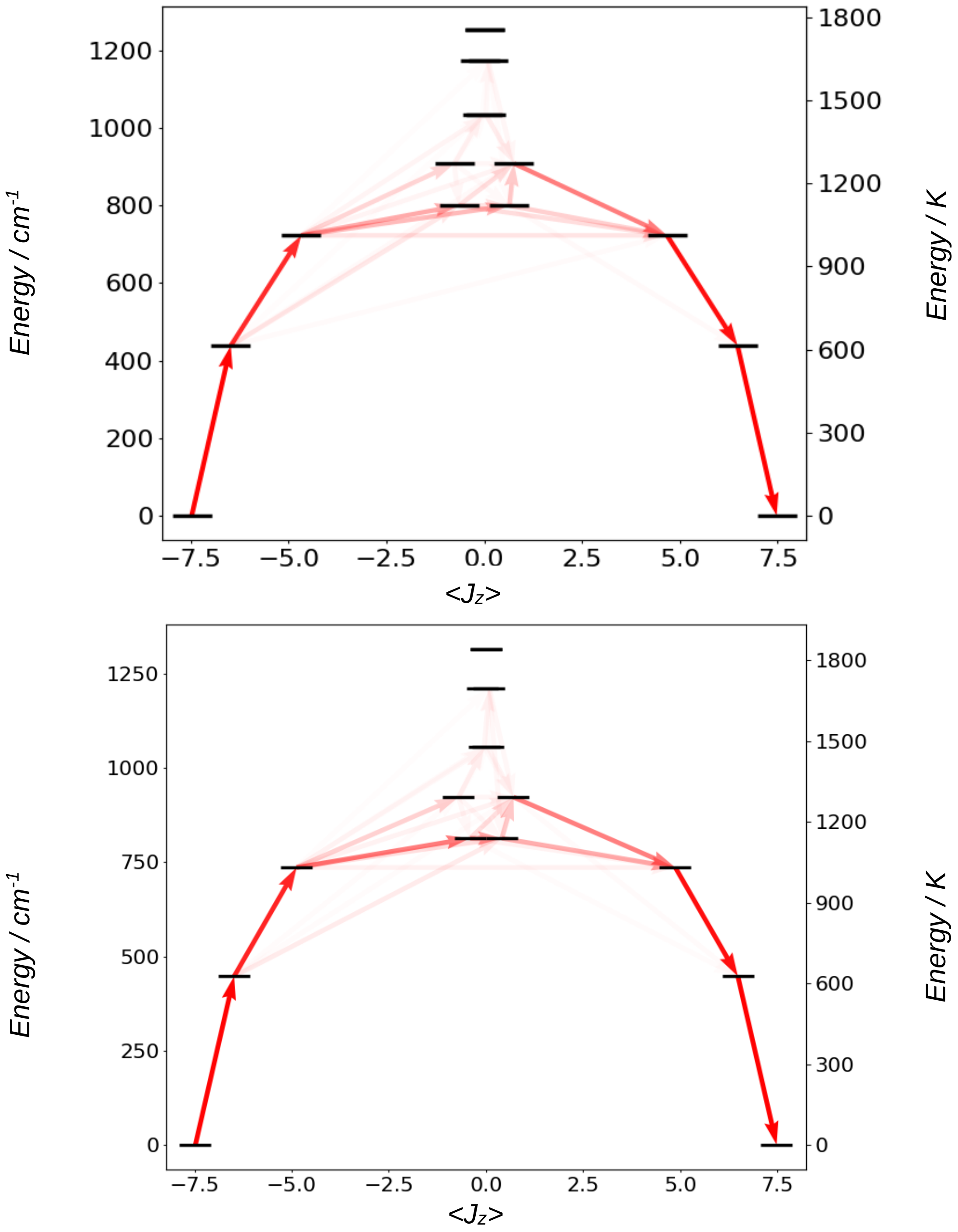

Figure S23. CASSCF-SO-calculated energy diagram of the ground-state multiplet for **2** (top) and **3** (bottom) indicating the zero-field magnetic transition propensities obtained from the average of the three Cartesian transition magnetic moment operators. The opacity of each arrow is proportional to the normalised transition propensity.

Table S6: *Ab initio* results for the J = 15/2 multiplet of Dy(III) in **1**, **2** and **3**.

	***Ab initio* Energy (cm^{-1})**	g_x	g_y	g_z	**g_z Angle (º)**	**Wavefunction**
**1**	0.00	0.00	0.00	19.86	--	99.2%|±15/2⟩
	414.32	0.02	0.02	16.98	0.78	98.3%|±13/2⟩
	689.40	0.96	2.25	13.11	7.38	86.3%|±11/2⟩ + 3.5%|±7/2⟩ + 6.8%|±3/2⟩ + 1.6%|±1/2⟩
	763.94	1.83	4.40	13.26	86.37	8.1%|±11/2⟩ + 20.2%|±9/2⟩ + 5.2%|±7/2⟩ + 19.6%|±5/2⟩ + 14.2%|±3/2⟩ + 32.6%|±1/2⟩
	860.54	3.58	5.10	9.26	87.85	3.2%|±11/2⟩ + 54.1%|±9/2⟩ + 19%|±7/2⟩ + 2.6%|±5/2⟩ + 18.1%|±3/2⟩ + 2.8%|±1/2⟩
	962.24	0.19	0.73	13.68	88.57	21.4%|±9/2⟩ + 45.5%|±7/2⟩ + 24.5%|±5/2⟩ + 7.5%|±1/2⟩
	1073.46	0.32	0.44	16.57	89.16	3.2%|±9/2⟩ + 24.9%|±7/2⟩ + 38.4%|±5/2⟩ + 27%|±3/2⟩ + 5.9%|±1/2⟩
	1113.22	0.04	0.13	19.13	87.44	1.8%|±7/2⟩ + 13.5%|±5/2⟩ + 33.7%|±3/2⟩ + 49.6%|±1/2⟩
**2**	0.00	0.00	0.00	19.87	--	97.9%|±15/2⟩ + 2%|±13/2⟩
	437.24	0.01	0.01	16.99	1.54	1.8%|±15/2⟩ + 95.1%|±13/2⟩ + 2.6%|±11/2⟩
	722.14	1.09	2.59	13.01	16.25	1.8%|±13/2⟩ + 80.3%|±11/2⟩ + 6.8%|±7/2⟩ + 1.5%|±5/2⟩ + 7%|±3/2⟩ + 2.1%|±1/2⟩
	799.26	1.86	4.65	12.15	82.20	9.7%|±11/2⟩ + 21.9%|±9/2⟩ + 3.6%|±7/2⟩ + 21.1%|±5/2⟩ + 13.4%|±3/2⟩ + 29.7%|±1/2⟩
	906.73	3.13	5.05	9.52	88.64	4.9%|±11/2⟩ + 50%|±9/2⟩ + 17.7%|±7/2⟩ + 3.5%|±5/2⟩ + 19.3%|±3/2⟩ + 4.5%|±1/2⟩
	1033.04	0.45	0.74	13.74	89.15	1.8%|±11/2⟩ + 23%|±9/2⟩ + 44%|±7/2⟩ + 20%|±5/2⟩ + 10.7%|±1/2⟩
	1174.18	0.21	0.30	16.72	89.97	4.5%|±9/2⟩ + 25.2%|±7/2⟩ + 38.8%|±5/2⟩ + 26.2%|±3/2⟩ + 4.8%|±1/2⟩
	1250.97	0.02	0.07	19.41	88.17	2.6%|±7/2⟩ + 14.7%|±5/2⟩ + 33.7%|±3/2⟩ + 48.2%|±1/2⟩
**3**	0.00	0.00	0.00	19.87	--	98.3%|±15/2⟩ + 1.6%|±13/2⟩
	446.05	0.01	0.01	17.00	1.88	1.5%|±15/2⟩ + 96.2%|±13/2⟩ + 1.9%|±11/2⟩
	735.66	1.10	2.63	12.97	14.73	1.3%|±13/2⟩ + 82.5%|±11/2⟩ + 6.7%|±7/2⟩ + 1.1%|±5/2⟩ + 6.2%|±3/2⟩ + 1.9%|±1/2⟩
	813.16	1.89	4.70	12.26	85.36	8.8%|±11/2⟩ + 20.8%|±9/2⟩ + 3.6%|±7/2⟩ + 22.1%|±5/2⟩ + 14.3%|±3/2⟩ + 30.1%|±1/2⟩
	921.49	3.04	4.95	9.59	90.00	4.6%|±11/2⟩ + 50.2%|±9/2⟩ + 17.8%|±7/2⟩ + 3.8%|±5/2⟩ + 19.6%|±3/2⟩ + 3.9%|±1/2⟩
	1055.76	0.44	0.73	13.74	90.00	1.8%|±11/2⟩ + 23.7%|±9/2⟩ + 43.2%|±7/2⟩ + 18.8%|±5/2⟩ + 12.1%|±1/2⟩
	1211.77	0.18	0.25	16.65	90.00	4.9%|±9/2⟩ + 26%|±7/2⟩ + 39.2%|±5/2⟩ + 25.8%|±3/2⟩ + 3.8%|±1/2⟩
	1314.32	0.02	0.05	19.48	90.00	2.7%|±7/2⟩ + 14.8%|±5/2⟩ + 33.9%|±3/2⟩ + 48.3%|±1/2⟩

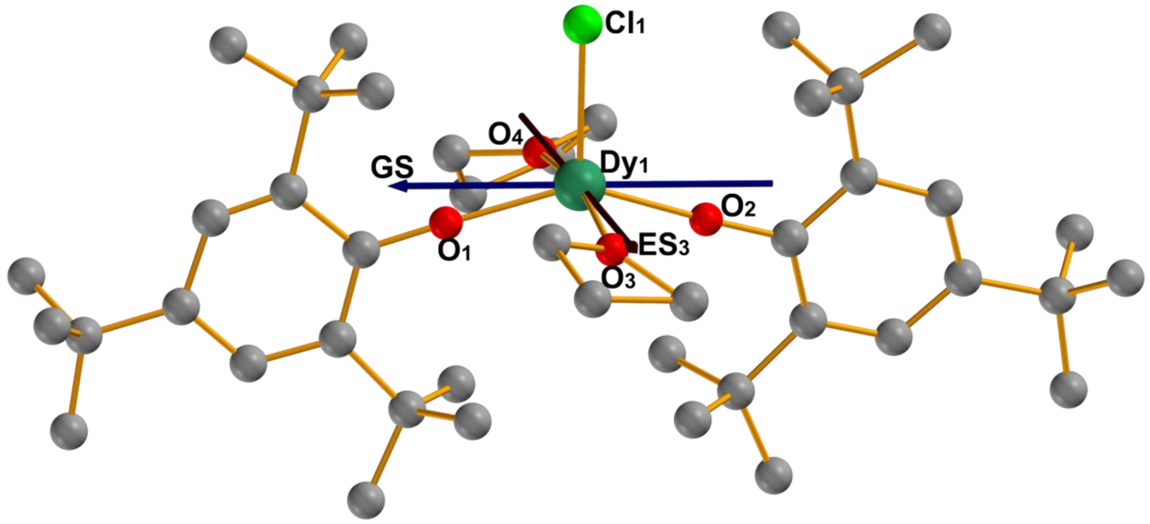

Figure S24: View of the [Dy(OMes*)$_2$(THF)$_2$Cl] molecular structure in **1** with the CASSCF-SO calculated magnetic axis (Gz vector) shown as blue arrow for the ground state and maroon arrow for the fourth state (Dy turquoise, Cl green, O red, C grey). H atoms are omitted for clarity.

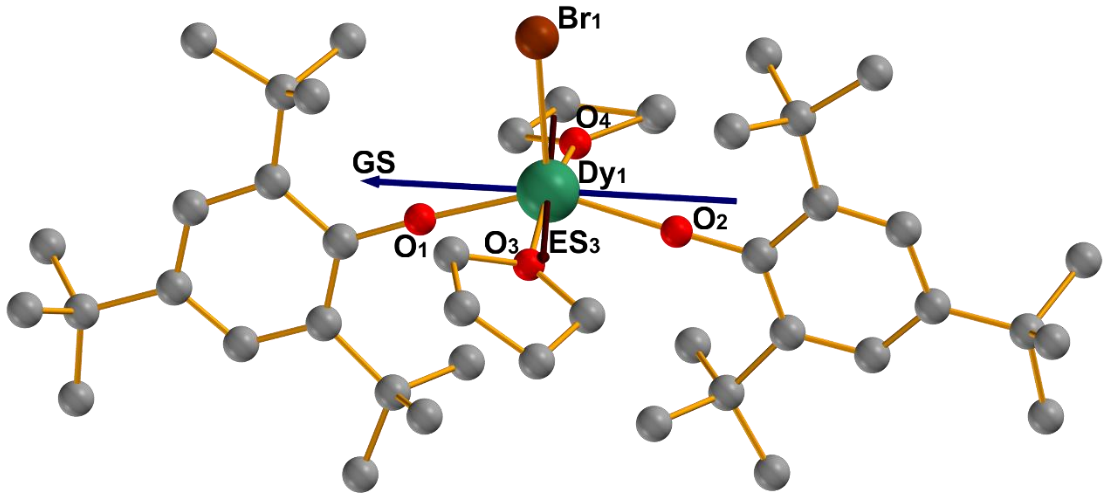

Figure S25: View of the [Dy(OMes*)$_2$(THF)$_2$Br] molecular structure in **2** with the CASSCF-SO calculated magnetic axis (Gz vector) shown as blue arrow for the ground state and maroon arrow for the fourth state (Dy turquoise, Cl green, O red, C grey). H atoms are omitted for clarity.

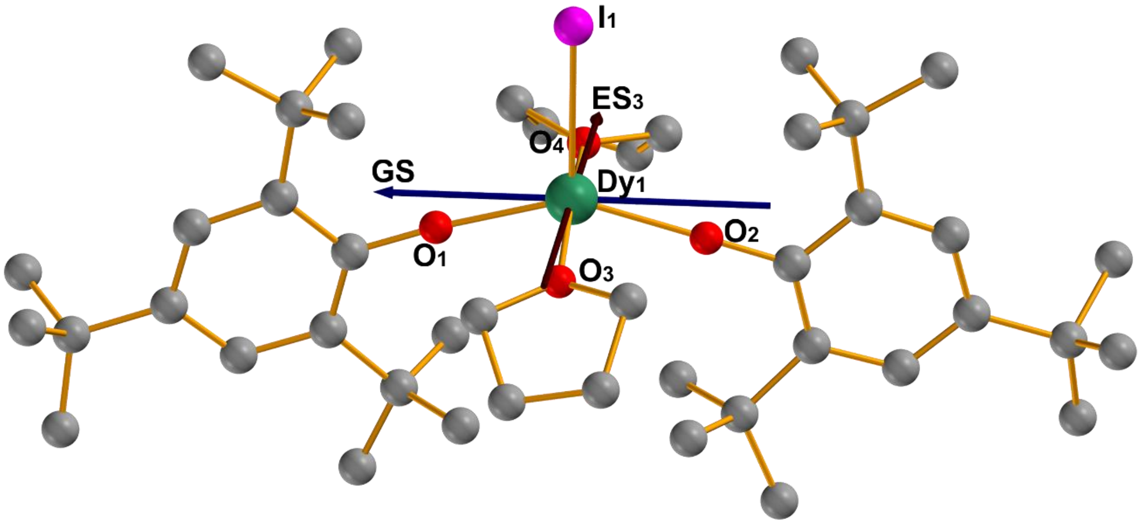

Figure S26: View of the [Dy(OMes*)$_2$(THF)$_2$I] molecular structure in **3** with the CASSCF-SO calculated magnetic axis (Gz vector) shown as blue arrow for the ground state and maroon arrow for the fourth state (Dy turquoise, Cl green, O red, C grey). H atoms are omitted for clarity.

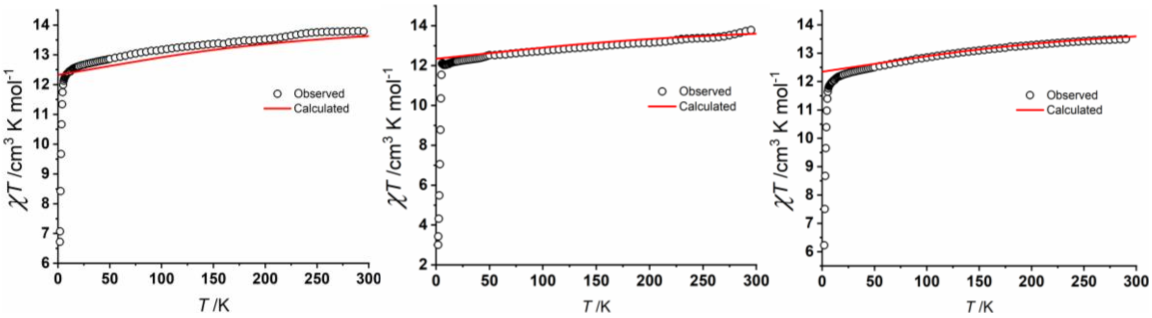

Figure S27. Experimental (black circles) and the CASSCF-SO calculated (red line) temperature dependence of the χT product between 0 and 300 K **1 (**left), **2** (centre) and **3** (right). χ is defined as magnetic susceptibility equal to M/H per mole. The experimental χT product is observed at 0.1 T field. The plots show the experimental and theoretical agreement for compounds **1**, **2**, and **3**.

Table S7: Average transition magnetic moment elements between the states on **1**.

	1	2	3	4	5	6	7	8	9	10	11	12	13	14	15	16
1		0.0000	3.6330	0.0028	0.0002	0.0067	0.0065	0.0027	0.0126	0.0034	0.0034	0.0032	0.0004	0.0016	0.0003	0.0003
2	0.0000		0.0028	3.6330	0.0067	0.0002	0.0027	0.0065	0.0034	0.0126	0.0032	0.0034	0.0016	0.0004	0.0003	0.0003
3	3.6330	0.0028		0.0621	0.0093	6.0570	0.0352	0.4188	0.0133	0.1460	0.0847	0.0158	0.0126	0.0193	0.0047	0.0051
4	0.0028	3.6330	0.0621		6.0570	0.0093	0.4188	0.0352	0.1460	0.0133	0.0158	0.0847	0.0193	0.0126	0.0051	0.0047
5	0.0002	0.0067	0.0093	6.0570		0.4284	2.1340	5.4030	0.4023	2.9740	0.8196	0.0273	0.0192	0.1976	0.0319	0.0231
6	0.0067	0.0002	6.0570	0.0093	0.4284		5.4030	2.1340	2.9740	0.4023	0.0273	0.8196	0.1976	0.0192	0.0231	0.0319
7	0.0065	0.0027	0.0352	0.4188	2.1340	5.4030		9.3600	3.9620	4.2700	0.0927	0.0559	0.0339	0.0708	0.0463	0.0330
8	0.0027	0.0065	0.4188	0.0352	5.4030	2.1340	9.3600		4.2700	3.9620	0.0559	0.0927	0.0708	0.0339	0.0330	0.0463
9	0.0126	0.0034	0.0133	0.1460	0.4023	2.9740	3.9620	4.2700		6.1620	5.7790	4.0630	0.0171	0.1405	0.0928	0.2192
10	0.0034	0.0126	0.1460	0.0133	2.9740	0.4023	4.2700	3.9620	6.1620		4.0630	5.7790	0.1405	0.0171	0.2192	0.0928
11	0.0034	0.0032	0.0847	0.0158	0.8196	0.0273	0.0927	0.0559	5.7790	4.0630		11.7600	3.9770	2.5750	0.1991	0.1295
12	0.0032	0.0034	0.0158	0.0847	0.0273	0.8196	0.0559	0.0927	4.0630	5.7790	11.7600		2.5750	3.9770	0.1295	0.1991
13	0.0004	0.0016	0.0126	0.0193	0.0192	0.1976	0.0339	0.0708	0.0171	0.1405	3.9770	2.5750		18.7100	1.7010	3.1010
14	0.0016	0.0004	0.0193	0.0126	0.1976	0.0192	0.0708	0.0339	0.1405	0.0171	2.5750	3.9770	18.7100		3.1010	1.7010
15	0.0003	0.0003	0.0047	0.0051	0.0319	0.0231	0.0463	0.0330	0.0928	0.2192	0.1991	0.1295	1.7010	3.1010		19.5400
16	0.0003	0.0003	0.0051	0.0047	0.0231	0.0319	0.0330	0.0463	0.2192	0.0928	0.1295	0.1991	3.1010	1.7010	19.5400	

Table S8: Average transition magnetic moment elements between the states on **2**.

	**1**	**2**	**3**	**4**	**5**	**6**	**7**	**8**	**9**	**10**	**11**	**12**	**13**	**14**	**15**	**16**
**1**		0.0000	3.6000	0.0000	0.0090	0.0000	0.0060	0.0020	0.0120	0.0030	0.0020	0.0040	0.0010	0.0010	0.0000	0.0000
**2**	0.0000		0.0000	3.6000	0.0000	0.0090	0.0020	0.0060	0.0030	0.0120	0.0040	0.0020	0.0010	0.0010	0.0000	0.0000
**3**	3.6000	0.0000		0.0000	5.9370	0.0030	0.0520	0.4730	0.0130	0.1890	0.0920	0.0110	0.0210	0.0090	0.0020	0.0030
**4**	0.0000	3.6000	0.0000		0.0030	5.9370	0.4730	0.0520	0.1890	0.0130	0.0110	0.0920	0.0090	0.0210	0.0030	0.0020
**5**	0.0090	0.0000	5.9370	0.0030		0.4960	4.1200	3.9950	2.2520	0.9300	0.0840	0.6430	0.0110	0.1560	0.0120	0.0130
**6**	0.0000	0.0090	0.0030	5.9370	0.4960		3.9950	4.1200	0.9300	2.2520	0.6430	0.0840	0.1560	0.0110	0.0130	0.0120
**7**	0.0060	0.0020	0.0520	0.4730	4.1200	3.9950		2.5650	3.2810	5.4120	0.1000	0.0250	0.0470	0.0360	0.0100	0.0350
**8**	0.0020	0.0060	0.4730	0.0520	3.9950	4.1200	2.5650		5.4120	3.2810	0.0250	0.1000	0.0360	0.0470	0.0350	0.0100
**9**	0.0120	0.0030	0.0130	0.1890	2.2520	0.9300	3.2810	5.4120		7.3710	6.4860	3.2820	0.0710	0.0200	0.0850	0.0330
**10**	0.0030	0.0120	0.1890	0.0130	0.9300	2.2520	5.4120	3.2810	7.3710		3.2820	6.4860	0.0200	0.0710	0.0330	0.0850
**11**	0.0020	0.0040	0.0920	0.0110	0.0840	0.6430	0.1000	0.0250	6.4860	3.2820		12.3400	2.8460	4.1350	0.0430	0.0450
**12**	0.0040	0.0020	0.0110	0.0920	0.6430	0.0840	0.0250	0.1000	3.2820	6.4860	12.3400		4.1350	2.8460	0.0450	0.0430
**13**	0.0010	0.0010	0.0210	0.0090	0.0110	0.1560	0.0470	0.0360	0.0710	0.0200	2.8460	4.1350		18.8500	1.2290	3.1390
**14**	0.0010	0.0010	0.0090	0.0210	0.1560	0.0110	0.0360	0.0470	0.0200	0.0710	4.1350	2.8460	18.8500		3.1390	1.2290
**15**	0.0000	0.0000	0.0020	0.0030	0.0120	0.0130	0.0100	0.0350	0.0850	0.0330	0.0430	0.0450	1.2290	3.1390		25.4000
**16**	0.0000	0.0000	0.0030	0.0020	0.0130	0.0120	0.0350	0.0100	0.0330	0.0850	0.0450	0.0430	3.1390	1.2290	25.4000	

Table S9: Average transition magnetic moment elements between the states on **3**.

	**1**	**2**	**3**	**4**	**5**	**6**	**7**	**8**	**9**	**10**	**11**	**12**	**13**	**14**	**15**	**16**
**1**		0.0000	0.1074	4.3350	0.0107	0.0002	0.0020	0.0082	0.0044	0.0151	0.0057	0.0027	0.0016	0.0008	0.0004	0.0000
**2**	0.0000		4.3350	0.1074	0.0002	0.0107	0.0082	0.0020	0.0151	0.0044	0.0027	0.0057	0.0008	0.0016	0.0000	0.0004
**3**	0.1074	4.3350		2.3510	0.1809	7.0660	0.0633	0.6639	0.0229	0.2359	0.1175	0.0111	0.0102	0.0267	0.0055	0.0015
**4**	4.3350	0.1074	2.3510		7.0660	0.1809	0.6639	0.0633	0.2359	0.0229	0.0111	0.1175	0.0267	0.0102	0.0015	0.0055
**5**	0.0107	0.0002	0.1809	7.0660		0.7021	4.7250	5.6910	1.2480	2.5330	0.8487	0.0369	0.0105	0.1943	0.0160	0.0136
**6**	0.0002	0.0107	7.0660	0.1809	0.7021		5.6910	4.7250	2.5330	1.2480	0.0369	0.8487	0.1943	0.0105	0.0136	0.0160
**7**	0.0020	0.0082	0.0633	0.6639	4.7250	5.6910		4.0520	4.2730	6.5610	0.1265	0.0394	0.0345	0.0670	0.0088	0.0470
**8**	0.0082	0.0020	0.6639	0.0633	5.6910	4.7250	4.0520		6.5610	4.2730	0.0394	0.1265	0.0670	0.0345	0.0470	0.0088
**9**	0.0044	0.0151	0.0229	0.2359	1.2480	2.5330	4.2730	6.5610		9.0710	7.9340	4.1030	0.0200	0.0891	0.1060	0.0381
**10**	0.0151	0.0044	0.2359	0.0229	2.5330	1.2480	6.5610	4.2730	9.0710		4.1030	7.9340	0.0891	0.0200	0.0381	0.1060
**11**	0.0057	0.0027	0.1175	0.0111	0.8487	0.0369	0.1265	0.0394	7.9340	4.1030		15.7900	5.4970	3.1220	0.1059	0.0002
**12**	0.0027	0.0057	0.0111	0.1175	0.0369	0.8487	0.0394	0.1265	4.1030	7.9340	15.7900		3.1220	5.4970	0.0002	0.1059
**13**	0.0016	0.0008	0.0102	0.0267	0.0105	0.1943	0.0345	0.0670	0.0200	0.0891	5.4970	3.1220		23.3300	1.1790	4.1950
**14**	0.0008	0.0016	0.0267	0.0102	0.1943	0.0105	0.0670	0.0345	0.0891	0.0200	3.1220	5.4970	23.3300		4.1950	1.1790
**15**	0.0004	0.0000	0.0055	0.0015	0.0160	0.0136	0.0088	0.0470	0.1060	0.0381	0.1059	0.0002	1.1790	4.1950		32.0600
**16**	0.0000	0.0004	0.0015	0.0055	0.0136	0.0160	0.0470	0.0088	0.0381	0.1060	0.0002	0.1059	4.1950	1.1790	32.0600	

Table S10: Crystal field parameters in **1**, **2** and **3**.

k	*q*	*B*(*k*,*q*) **1**	*B*(*k*,*q*) **2**	*B*(*k*,*q*) **3**
2	-2	1.50E+00	-1.02E-01	-4.01E-04
2	-1	3.39E-01	1.49E+00	1.43E+00
2	0	-5.58E+00	-6.11E+00	-6.34E+00
2	1	-5.62E-01	6.39E-02	9.09E-05
2	2	-1.90E+00	3.45E+00	3.89E+00
4	-4	2.63E-02	-3.86E-03	5.92E-06
4	-3	-1.60E-02	2.93E-02	2.57E-02
4	-2	-1.28E-02	-1.19E-04	3.83E-06
4	-1	1.23E-03	2.10E-02	1.92E-02
4	0	-9.27E-03	-8.98E-03	-8.87E-03
4	1	-1.03E-02	-2.67E-03	2.03E-06
4	2	1.79E-02	-2.17E-02	-2.37E-02
4	3	2.23E-03	-1.75E-04	4.53E-06
4	4	-4.02E-03	-2.43E-02	-2.17E-02
6	-6	-1.23E-04	-1.55E-05	6.12E-08
6	-5	1.38E-04	2.68E-04	2.44E-04
6	-4	1.23E-04	-1.84E-05	3.33E-08
6	-3	-7.86E-05	1.15E-04	1.14E-04
6	-2	-5.24E-05	-3.12E-06	9.05E-09
6	-1	1.56E-05	-1.92E-07	2.03E-05
6	0	9.30E-06	9.59E-06	8.94E-06
6	1	2.66E-06	1.13E-05	2.50E-09
6	2	5.81E-05	-6.50E-05	-5.43E-05
6	3	-5.52E-07	-2.10E-07	2.44E-08
6	4	-1.87E-05	-1.15E-04	-1.14E-04
6	5	5.68E-05	-5.32E-05	6.47E-08
6	6	-5.10E-05	-1.58E-04	-1.53E-04

7. Magnetism

Magnetic susceptibility measurements were performed on a Quantum Design SQUID MPMS-XL magnetometer and PPMS-II susceptometer housed at the Centre de Recherche Paul Pascal at temperatures between 1.8 and 400 K and *dc* magnetic fields ranging from -9 to +9 T. The *ac* magnetic susceptibility measurements were performed in an oscillating *ac* field of 1 to 6 Oe with frequencies between 10 and 10000 Hz in zero *dc*-field. The measurements were carried out on polycrystalline samples (12.4, 7.3, 9.1, 34.0 and 32.5 for **1**, 12.5 mg for **2**, 9.3 mg for **3** and 33.5 and 35.2 mg for **5%Dy@1-Y**) suspended in mineral oil (typically 7-19 mg) and introduced in a sealed polyethene bag (3 $\times$ 0.5 $\times$ 0.02 cm; typically, 13-31 mg) in a glovebox a controlled atmosphere of nitrogen or argon. Prior to the experiments, the field-dependent magnetisation was measured at 100 K on each sample in order to detect the presence of any bulk ferromagnetic impurities. As expected for paramagnetic or diamagnetic materials, a perfectly linear dependence of the magnetisation that extrapolates to zero at zero *dc* field was systematically observed; the samples appeared to be free of any ferromagnetic impurities. The magnetic susceptibilities were corrected for the sample holder, the mineral oil and the intrinsic diamagnetic contributions.

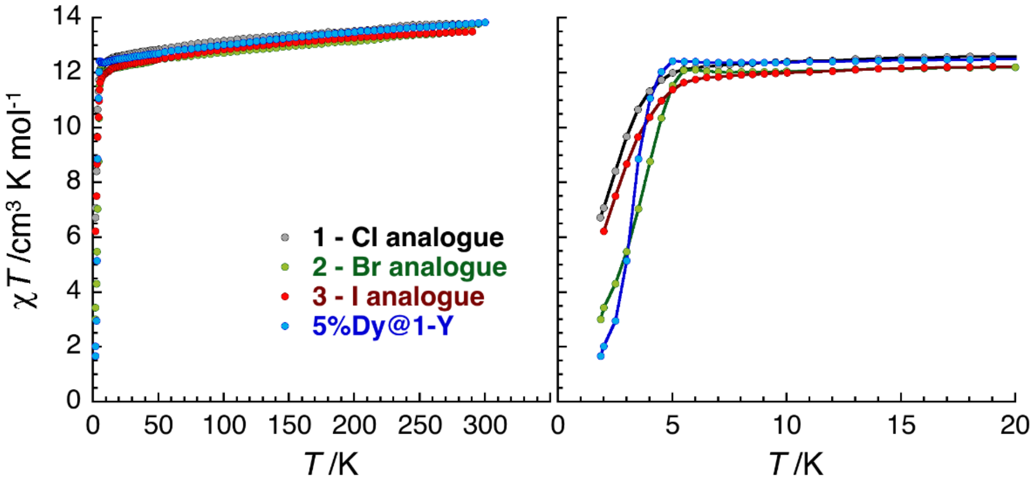

Figure S28. Temperature dependence of the χT product at 0.1 T between 0 and 300 K (left) and 0 and 20 K (right) for **1**, **2**, **3** and **5%Dy@1-Y** at 0.3 K/min. χ is defined as magnetic susceptibility equal to *M*/*H* per mole of complex except for **5%Dy@1-Y**, for which χ has been multiplied by 20, for clarity, according to the 5% Dy dilution. The χT products at 270, 100 and 1.85 K are 13.8, 13.2 and 6.72 cm^3 K/mol for **1**, 13.8, 13.2 and 6.72 cm^3 K/mol for **1**, 13.5, 12.7 and 3.01 cm^3 K/mol for **2**, 13.5, 12.8 and 6.22 cm^3 K/mol for **3** and 13.7, 13.0 and 1.66 cm^3 K/mol for **5%Dy@1-Y**.

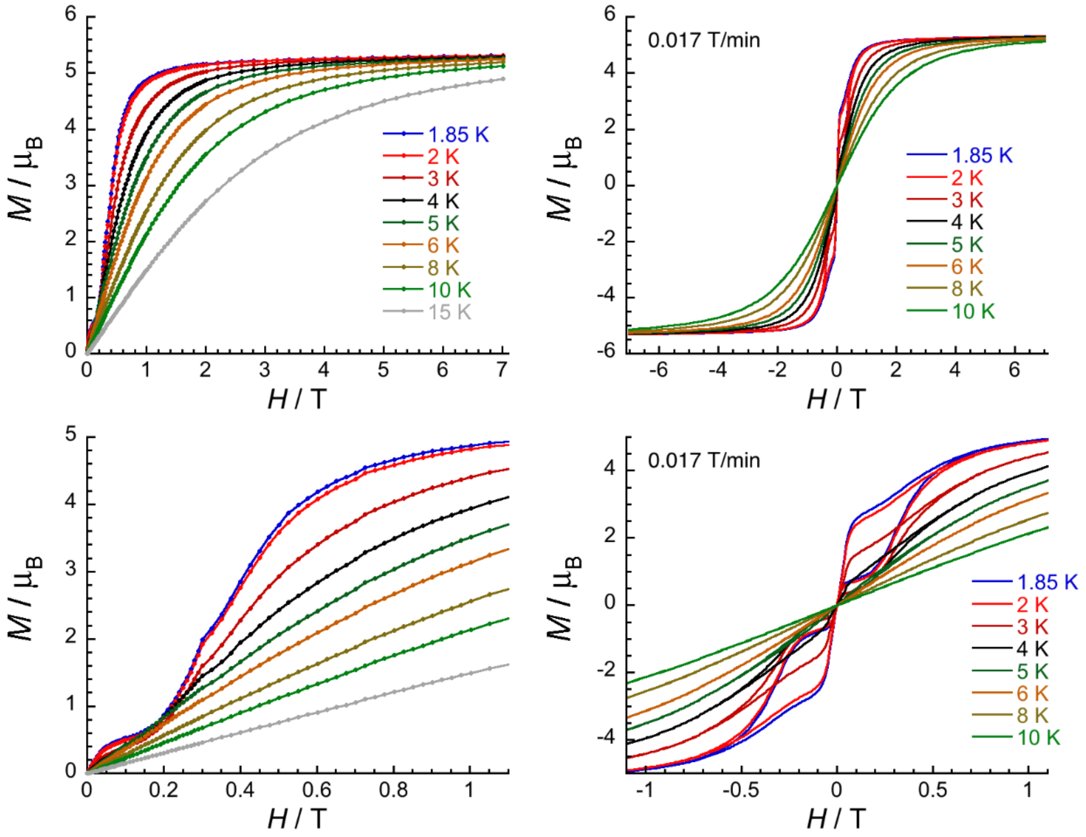

Figure S29. Field dependence of the magnetisation for **1**. On the left, the *M* vs *H* data between 1.85 and 15 K have been measured after a zero-field cooling from 20 K to the indicated temperature in order to obtain first magnetization curves between 0 and 7 T (top); a zoom of these data between 0 and 1.1 T is shown in the bottom part of the figure (field sweeping rates: 9 mT/min between 0 and 0.1 T, 18 mT/min between 0.1 and 0.35 T, 40 mT/min between 0.35 and 1 T, 84 mT/min between 1 and 2 T, 280 mT/min between 2 and 7 T). On the right, the *M* vs *H* data between 1.85 and 10 K have been measured at 17 mT/min after saturating the magnetisation at 7 T (*M*(1.85 K, 7 T) = 5.3 μ_B) in order to obtain magnetisation hysteresis curves between -7 and 7 T (top); a zoom of these data between -1.1 and 1.1 T is shown in the bottom part of the figure.

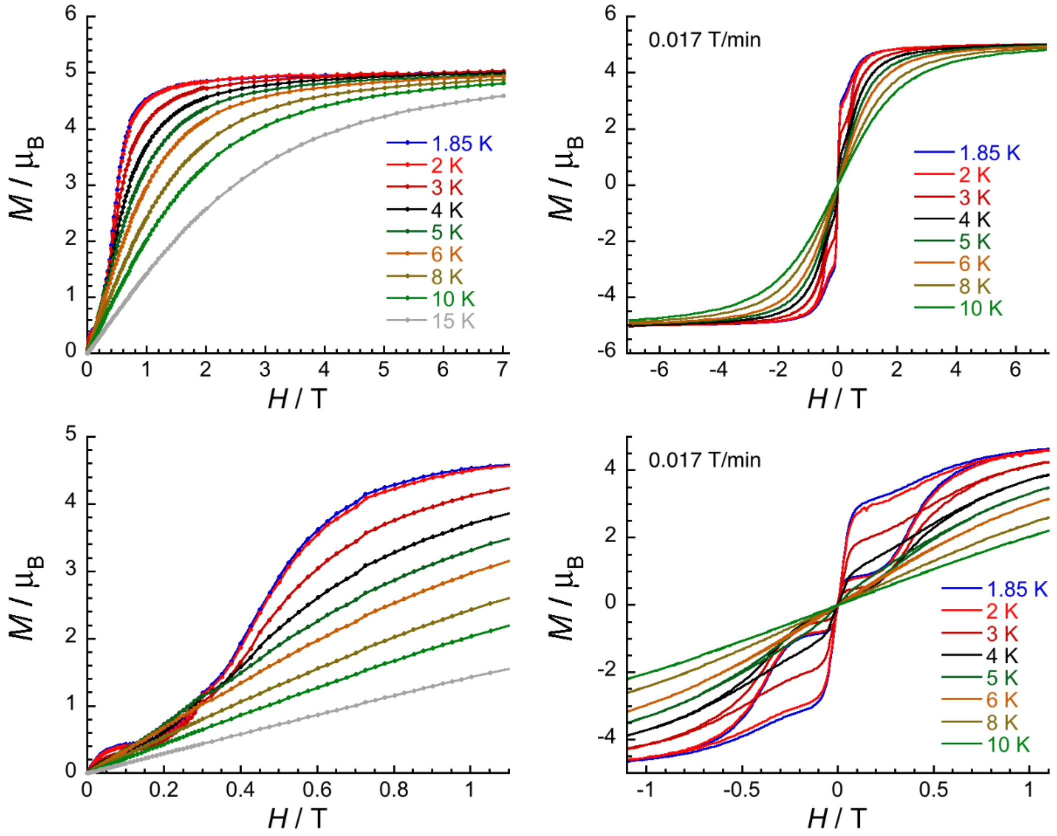

Figure S30. Field dependence of the magnetisation for **2**. On the left, the *M* vs *H* data between 1.85 and 15 K have been measured after a zero-field cooling from 20 K to the indicated temperature in order to obtain first magnetisation curves between 0 and 7 T (top); a zoom of these data between 0 and 1.1 T is shown in the bottom part of the figure (field sweeping rates: 9 mT/min between 0 and 0.1 T, 18 mT/min between 0.1 and 0.35 T, 40 mT/min between 0.35 and 1 T, 84 mT/min between 1 and 2 T, 280 mT/min between 2 and 7 T). On the right, the *M* vs *H* data between 1.85 and 10 K have been measured at 17 mT/min after saturating the magnetisation at 7 T (*M*(1.85 K, 7 T) = 5.0 μ_B) in order to obtain magnetisation hysteresis curves between -7 and 7 T (top); a zoom of these data between -1.1 and 1.1 T is shown in the bottom part of the figure.

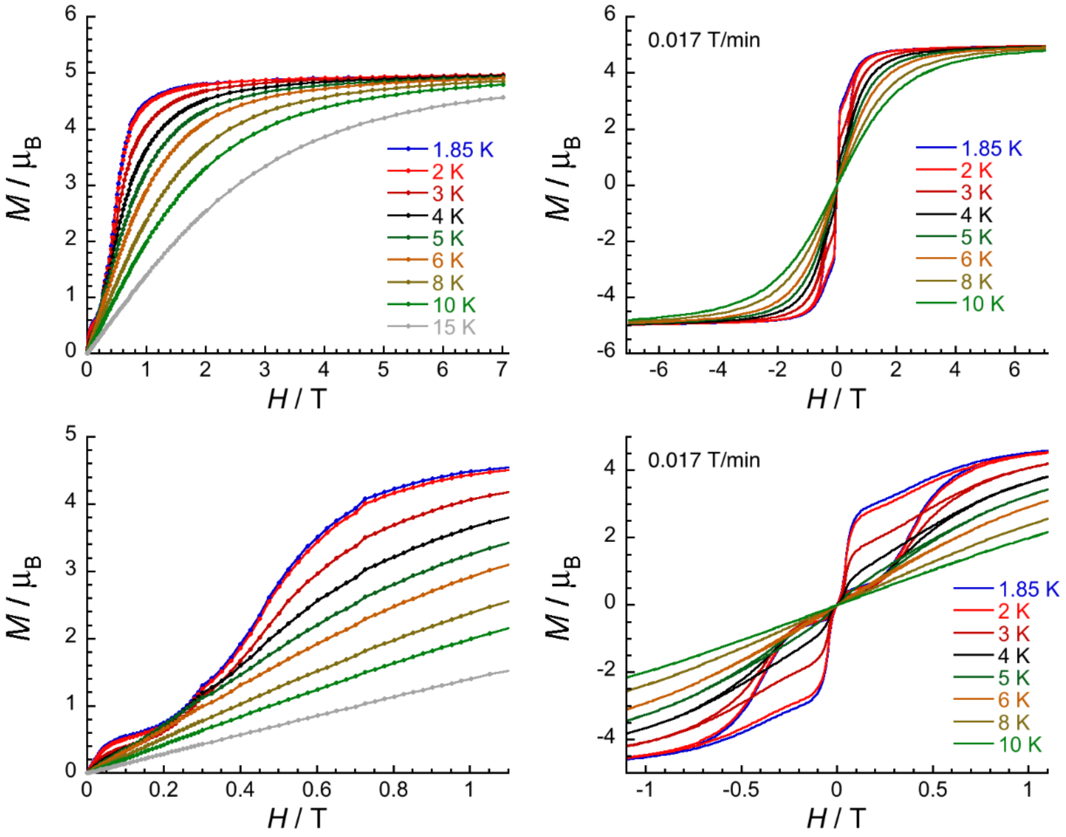

Figure S31. Field dependence of the magnetisation for **3**. On the left, the *M* vs *H* data between 1.85 and 15 K have been measured after a zero-field cooling from 20 K to the indicated temperature in order to obtain first magnetisation curves between 0 and 7 T (top); a zoom of these data between 0 and 1.1 T is shown in the bottom part of the figure (field sweeping rates: 9 mT/min between 0 and 0.1 T, 18 mT/min between 0.1 and 0.35 T, 40 mT/min between 0.35 and 1 T, 84 mT/min between 1 and 2 T, 280 mT/min between 2 and 7 T). On the right, the *M* vs *H* data between 1.85 and 10 K have been measured at 17 mT/min after saturating the magnetisation at 7 T (*M*(1.85 K, 7 T) = 5.0 μ_B) in order to obtain magnetisation hysteresis curves between -7 and 7 T (top); a zoom of these data between -1.1 and 1.1 T is shown in the bottom part of the figure.

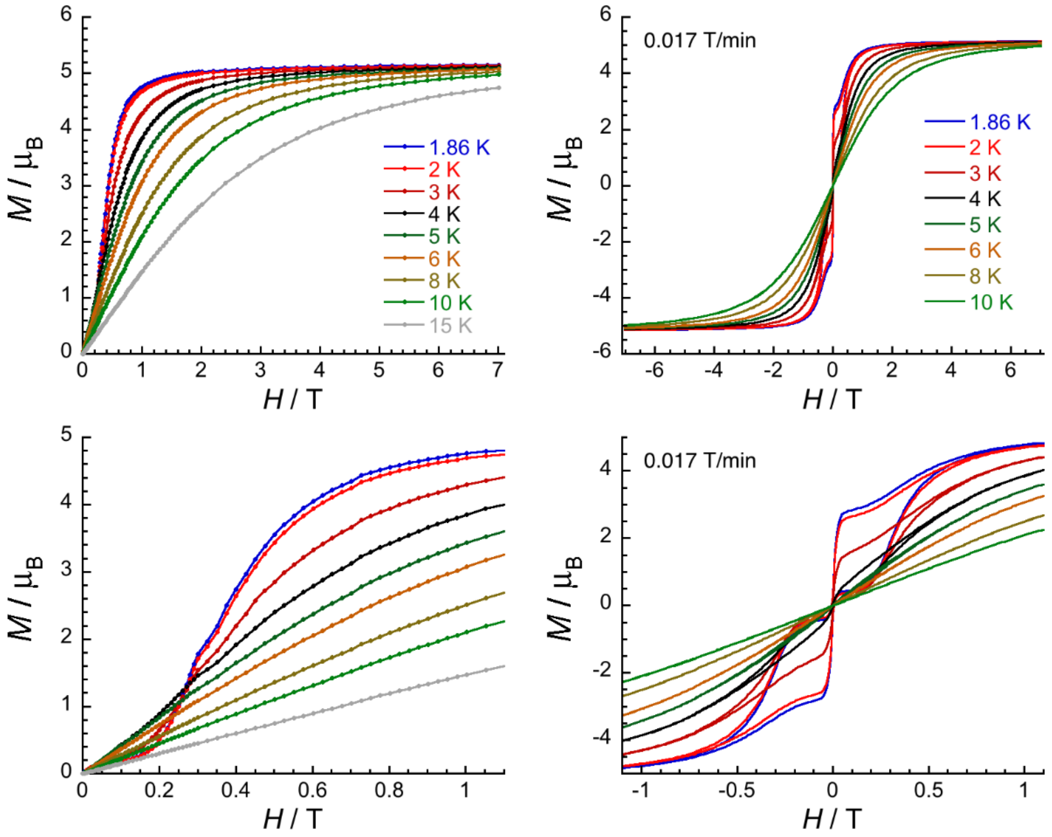

Figure S32. Field dependence of the magnetisation (multiplied by 20 according to the 5% Dy dilution for easier comparison with the other complexes) for **5%Dy@1-Y**. On the left, the *M* vs *H* data between 1.85 and 15 K have been measured after a zero-field cooling from 20 K to the indicated temperature in order to obtain first magnetisation curves between 0 and 7 T (top); a zoom of these data between 0 and 1.1 T is shown in the bottom part of the figure (field sweeping rates: 9 mT/min between 0 and 0.1 T, 18 mT/min between 0.1 and 0.35 T, 40 mT/min between 0.35 and 1 T, 84 mT/min between 1 and 2 T, 280 mT/min between 2 and 7 T). On the right, the *M* vs *H* data between 1.85 and 10 K have been measured at 17 mT/min after saturating the magnetisation at 7 T (M(1.85 K, 7 T) = 5.2 μ_B) in order to obtain magnetisation hysteresis curves between -7 and 7 T (top); a zoom of these data between -1.1 and 1.1 T is shown in the bottom part of the figure.

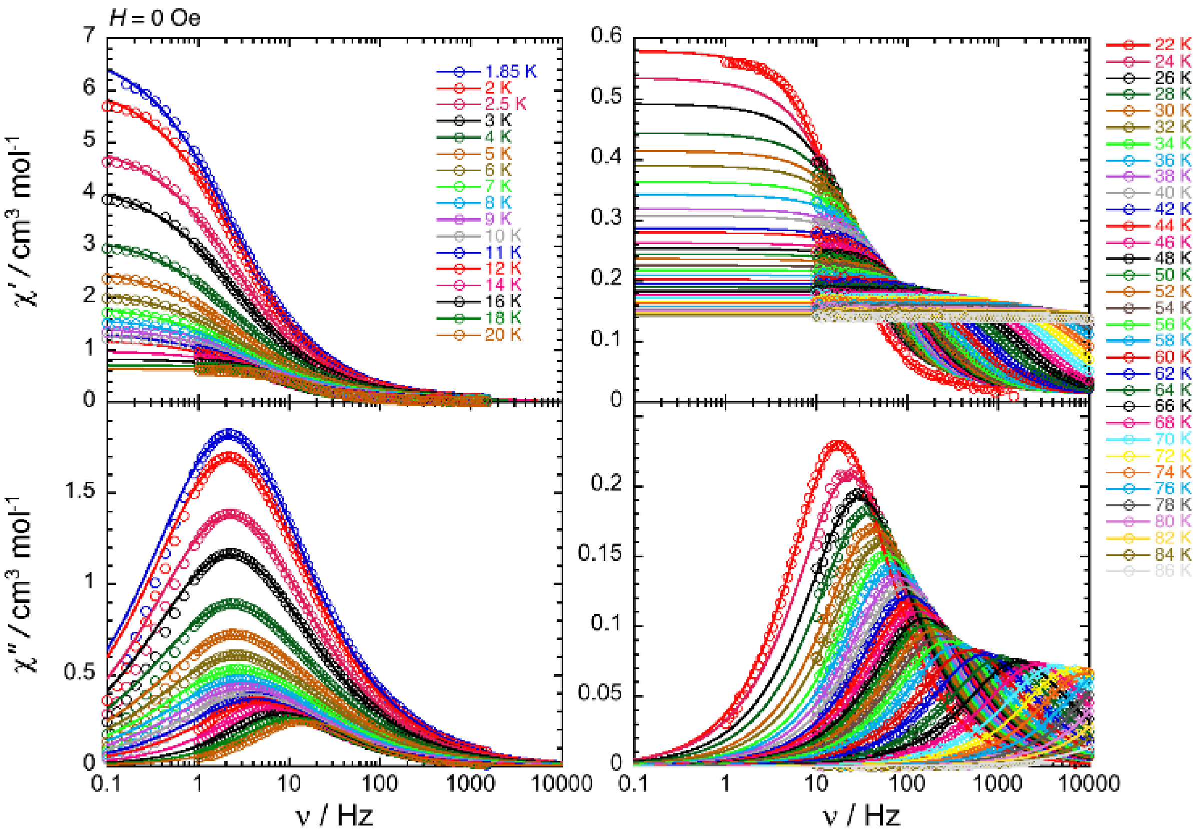

Figure 33. ac frequency dependences of the real (χ', top) and imaginary (χ'', bottom) parts of the ac susceptibility for **1**, between 1.85 and 20 K (left) and between 22 and 86 K (right) for ac frequencies between 0.1 and 10000 Hz (in a zero-dc field). Solid lines are the generalised Debye fit of the ac data used to extract the temperature dependence of the relaxation time.

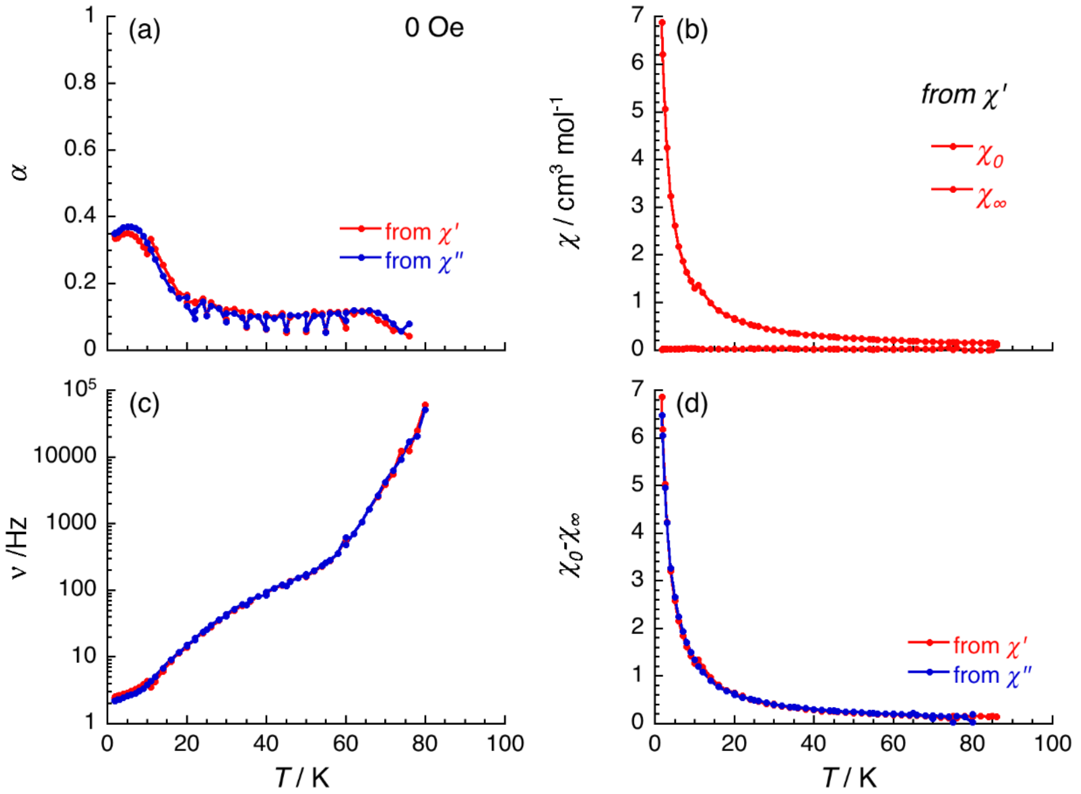

Figure S34. Field dependence of the parameters *α*, *ν*, χ_0, χ_∞ and χ_0-χ_∞, between 1.85 and 86 K in zero-dc field deduced from the generalised Debye fit of the frequency dependence of the real (χ') and imaginary (χ'') components of the ac susceptibility shown in Figure S33, for **1**.

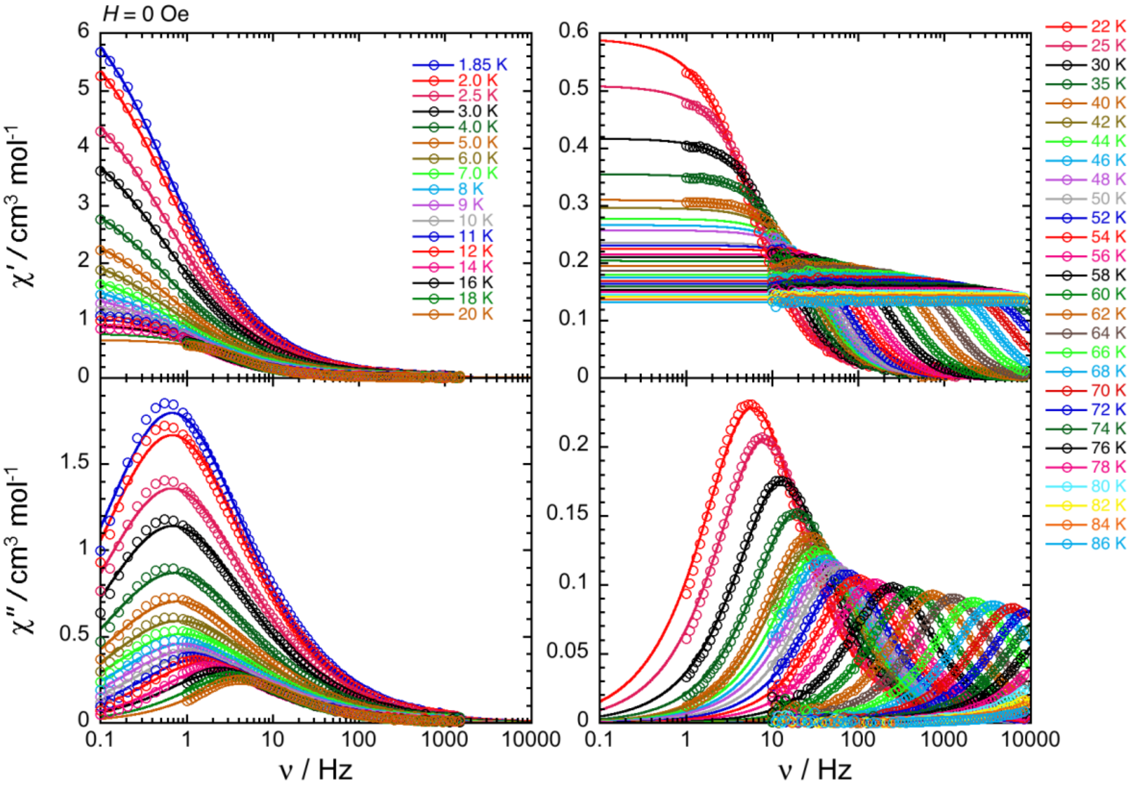

Figure S35. ac frequency dependences of the real (χ', top) and imaginary (χ'', bottom) parts of the ac susceptibility for **2**, between 1.85 and 20 K (left) and between 22 and 86 K (right) for ac frequencies between 0.1 and 10000 Hz (in a zero-dc field). Solid lines are the generalised Debye fit of the ac data used to extract the temperature dependence of the relaxation time.

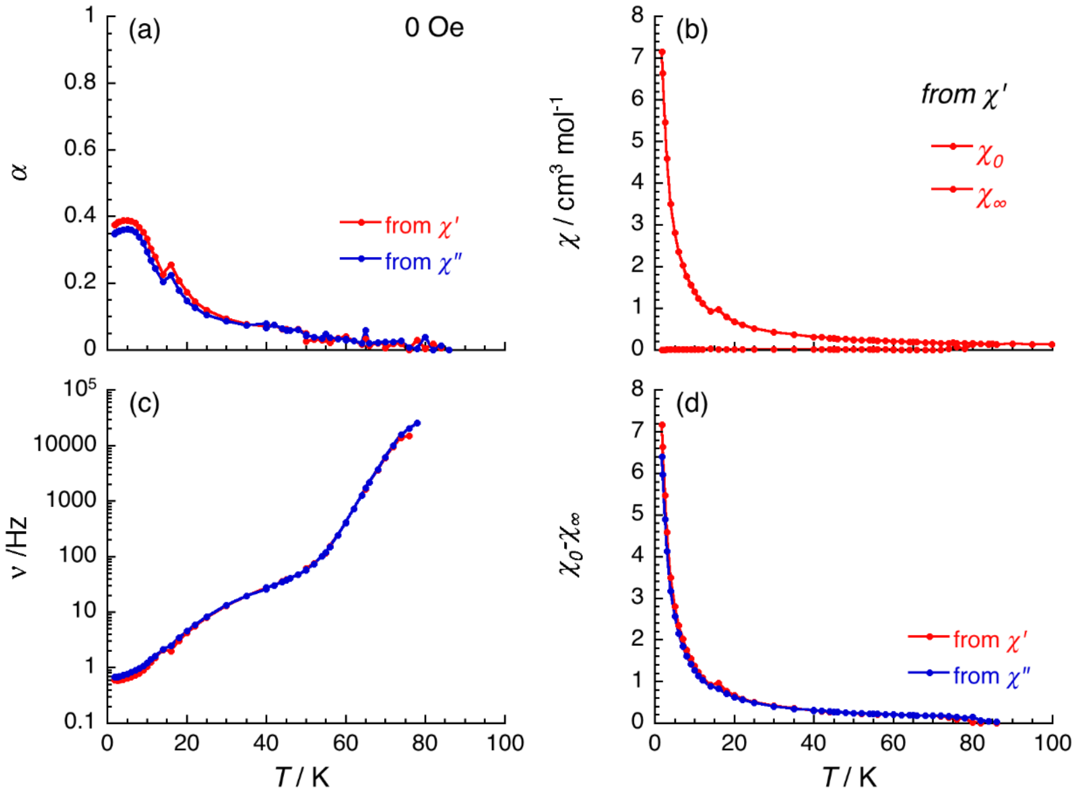

Figure S36. Field dependence of the parameters, α, ν, χ_0, χ_∞ and χ_0-χ_∞, between 1.85 and 86 K in zero-dc field deduced from the generalised Debye fit of the frequency dependence of the real (χ') and imaginary (χ'') components of the ac susceptibility shown in Figure S35, for **2**.

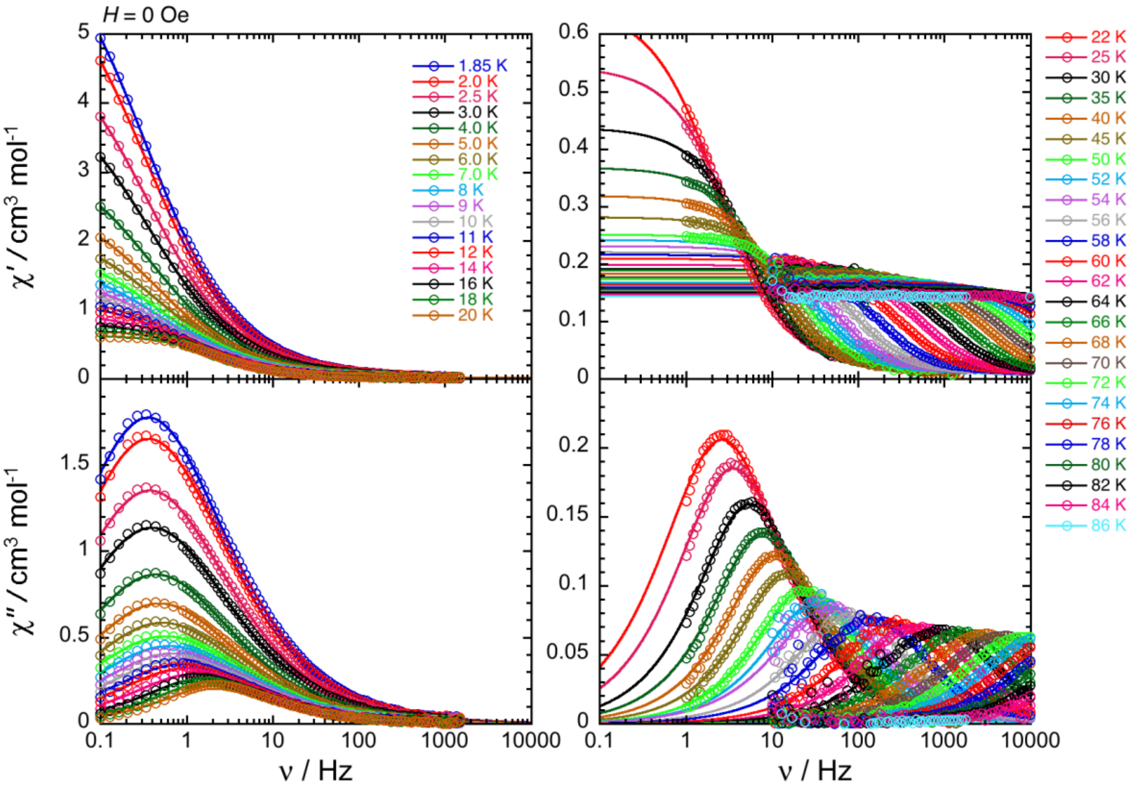

Figure S37. ac frequency dependences of the real (χ', top) and imaginary (χ'', bottom) parts of the ac susceptibility for **3**, between 1.85 and 20 K (left) and between 22 and 86 K (right) for ac frequencies between 0.1 and 10000 Hz (in a zero-dc field). Solid lines are the generalised Debye fit of the ac data used to extract the temperature dependence of the relaxation time.

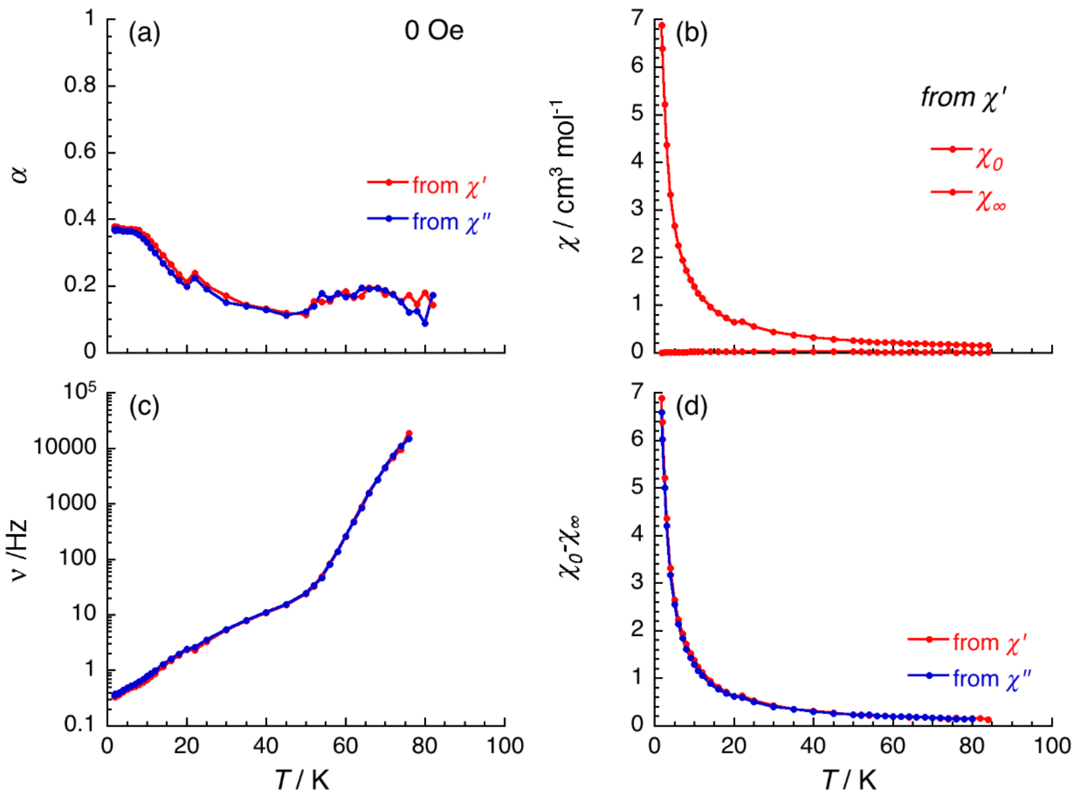

Figure S38. Field dependence of the parameters, α, v, χ_0, χ_∞ and χ_0-χ_∞, between 1.85 and 86 K in zero-dc field deduced from the generalised Debye fit of the frequency dependence of the real (χ') and imaginary (χ'') components of the ac susceptibility shown in Figure S37, for **3**.

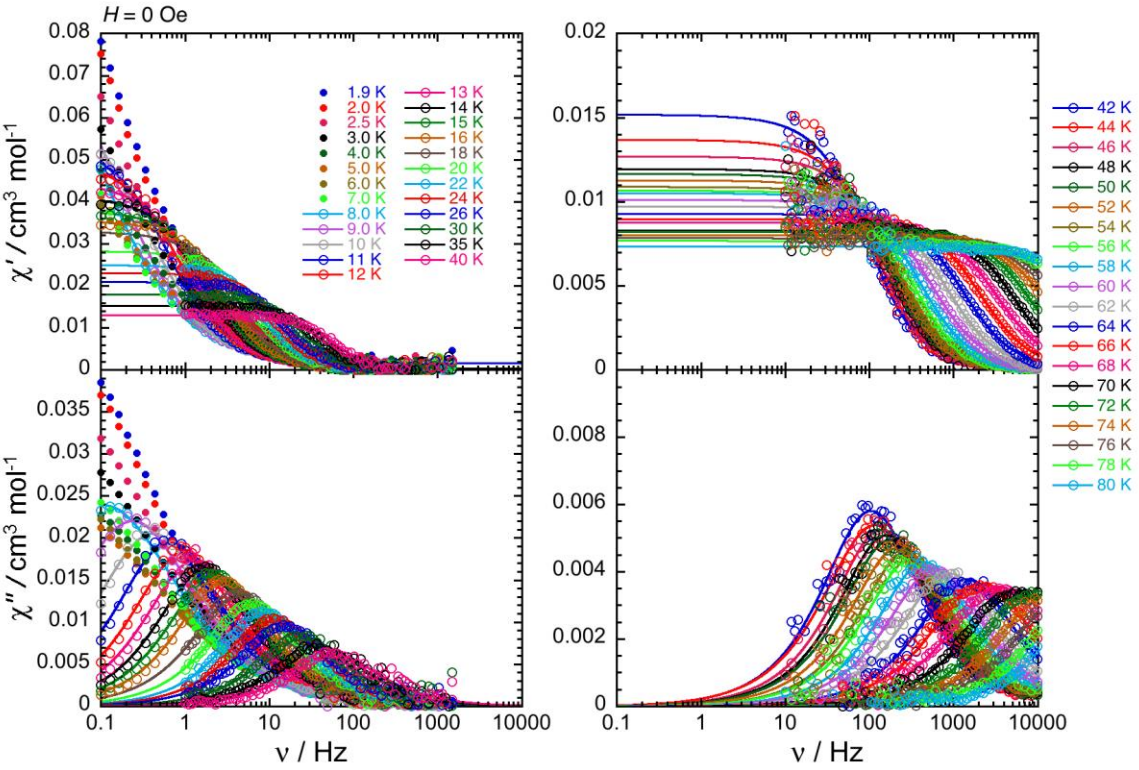

Figure S39. ac frequency dependences of the real (χ', top) and imaginary (χ'', bottom) parts of the ac susceptibility for **5%Dy@1-Y**, between 1.85 and 20 K (left) and between 22 and 86 K (right) for ac frequencies between 0.1 and 10000 Hz (in a zero-dc field). Solid lines are the generalised Debye fit of the ac data used to extract the temperature dependence of the relaxation time.

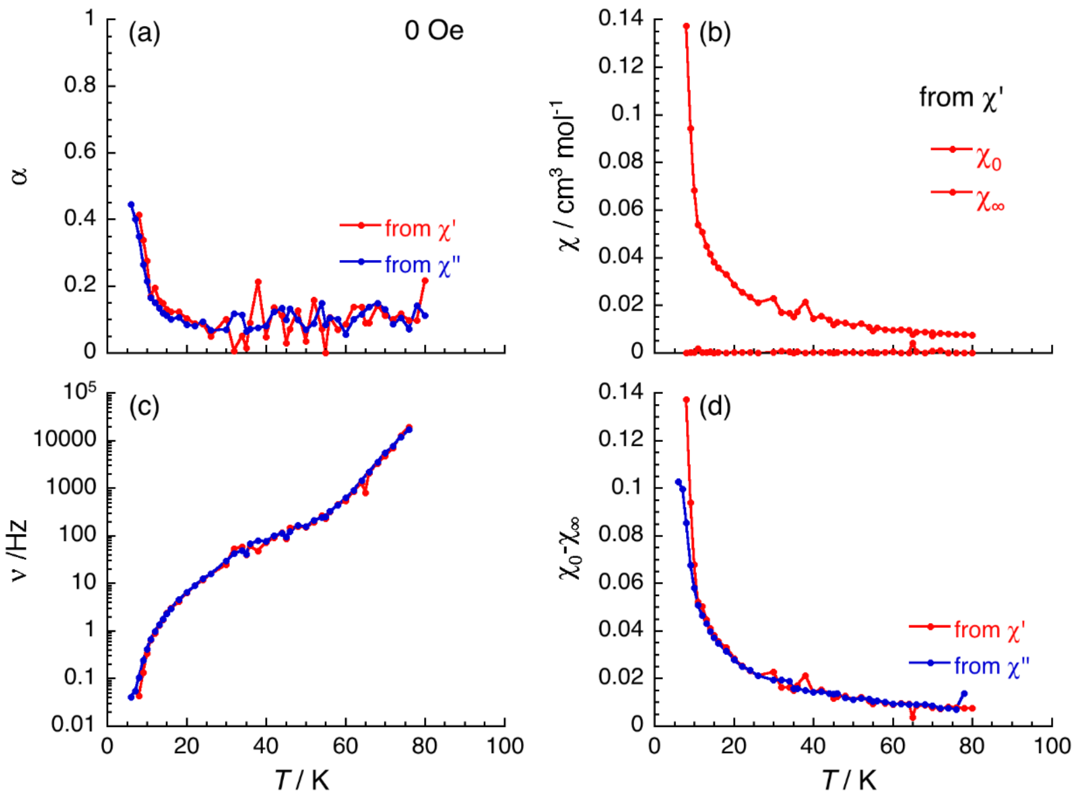

Figure S40. Field dependence of the parameters, *α*, *ν*, χ_0, χ_∞ and χ_0-χ_∞, between 1.85 and 86 K in zero-dc field deduced from the generalised Debye fit of the frequency dependence of the real (χ') and imaginary (χ'') components of the ac susceptibility shown in Figure S39, for **5%Dy@1-Y**.

Table S11 Recent selected top eight compounds with highest activation energy values and their relaxation parameters. (T_B is given for a 100 s characteristic time).

	**Δ/*k*$_B$ (K)**	**τ_0 (10^{-12} s)**	**C (s^{-1} K^{-n})**	***n***	**τ_{Raman}^{30K} (s)**	**τ_{QTM} (s)**	**T_B^{100s} (K)**	**Ref.**
$[Dy(Cp(Me)_5)(Cp(^iPr)_5)][B(C_6F_5)_4]$	2217	4.2	3.1×10^{-8}	3	1195	25000	65	[14]
$[Dy\{Cp(^iPr)_4(Me)\}_2][B(C_6F_5)_4]$	2112	4.01	1.57×10^{-6}	2.07	558	2452	62	[15]
$[Dy\{Cp(^iPr)_5\}_2][B(C_6F_5)_4]$	1919	11.8	8.04×10^{-7}	2.31	481	1187	59	[15]
$[Dy(Cp^{ttt})_2][B(C_6F_5)_4]$	1760	19.9	1.66×10^{-6}	2.15	402	—	53	[16]
$[Dy\{Cp(^iPr)_4(Et)\}_2][B(C_6F_5)_4]$	1986	7.79	3.36×10^{-8}	3.02	1030	447	59	[15]
$[Dy\{Cp(^iPr)_4\}_2][B(C_6F_5)_4]$	1848	3.39	2.27×10^{-5}	2	49	439	17	[15]
$[Dy(O^tBu)_2(py)_5][BPh_4]$	1815	1.17	1.0×10^{-6}	3.77	2.7	—	12	[17]
$[Dy\{P(CtBuCMe)_2\}_2][Al\{OC(CF_3)_3\}_4]$	1760	2.0	3.1×10^{-4}	1.1	77	—	24	[18]

8. References:

[1] S. K. Sur, *J. Magn. Reson.* **1989**, *82*, 169–173.

[2] D. F. Evans, *J. Chem. Soc.* **1959**, 2003.

[3] Agilent, *Technol. UK Ltd, Yarnton, Oxford, UK* **2014**, *44*, 1–53.

[4] G. M. Sheldrick, *Acta Crystallogr. Sect. A Found. Crystallogr.* **2008**, *64*, 112–122.

[5] G. M. Sheldrick, *Acta Crystallogr. Sect. C Struct. Chem.* **2015**, *71*, 3–8.

[6] O. V. Dolomanov, L. J. Bourhis, R. J. Gildea, J. A. K. Howard, H. Puschmann, *J. Appl. Crystallogr.* **2009**, *42*, 339–341.

[7] L. J. Farrugia, *J. Appl. Crystallogr.* **2012**, *45*, 849–854.

[8] Persistence of Vision Raytracer (Version 3.6), **2004**.

[9] S. Alvarez, P. Alemany, D. Casanova, J. Cirera, M. Llunell, D. Avnir, *Coord. Chem. Rev.* **2005**, *249*, 1693–1708.

[10] F. Aquilante, J. Autschbach, R. K. Carlson, L. F. Chibotaru, M. G. Delcey, L. De Vico, I. Fdez. Galván, N. Ferré, L. M. Frutos, L. Gagliardi, et al., *J. Comput. Chem.* **2016**, *37*, 506–541.

[11] B. O. Roos, R. Lindh, P. Å. Malmqvist, V. Veryazov, P. O. Widmark, *J. Phys. Chem. A* **2004**, *108*, 2851–2858.

[12] B. O. Roos, R. Lindh, P.-Å. Malmqvist, V. Veryazov, P.-O. Widmark, *J. Phys. Chem. A* **2005**, *109*, 6575–6579.

[13] L. Ungur, L. F. Chibotaru, *Chem. Eur. J.* **2017**, *23*, 3708–3718.

[14] F. S. Guo, B. M. Day, Y. C. Chen, M. L. Tong, A. Mansikkamäki, R. A. Layfield, *Science* **2018**, *362*, 1400–1403.

[15] K. R. McClain, C. A. Gould, K. Chakarawet, S. J. Teat, T. J. Groshens, J. R. Long, B. G. Harvey, *Chem. Sci.* **2018**, *9*, 8492–8503.

[16] C. A. P. Goodwin, F. Ortu, D. Reta, N. F. Chilton, D. P. Mills, *Nature* **2017**, *548*, 439–442.

[17] Y. S. Ding, N. F. Chilton, R. E. P. Winpenny, Y. Z. Zheng, *Angew. Chem. Int. Ed.* **2016**, *55*, 16071–16074.

[18] P. Evans, D. Reta, G. F. S. Whitehead, N. F. Chilton, D. P. Mills, *J. Am. Chem. Soc.* **2019**, *141*, 19935–19940.
